# Voice as objective biomarker of stress: association of speech features and cortisol

**DOI:** 10.1017/neu.2025.10037

**Published:** 2025-09-03

**Authors:** Felix Menne, Hali Lindsay, Johannes Tröger, Silke Paulmann, Alexandra König, Nadine Steinbach, Andreas Reif, Michael M. Plichta, Maren Schmidt-Kassow

**Affiliations:** 1 ki:elements GmbH, Saarbrücken, Germany; 2 Department of Psychology and Centre for Brain Science, University of Essex, Colchester, UK; 3 Centre Hospitalier et Universitaire, Clinique Gériatrique du Cerveau et du Mouvement, Centre Mémoire de Ressources et de Recherche, Université Côte d’Azur, Nice, France; 4 Department of Psychiatry, Psychosomatic Medicine and Psychotherapy, University Hospital, Goethe-University Frankfurt am Main, Frankfurt, Germany

**Keywords:** Voice, stress, physiological stress, psychological, speech, cortisol

## Abstract

**Objective:**

Cortisol is a well-established biomarker of stress, assessed through salivary or blood samples, which are intrusive and time-consuming. Speech, influenced by physiological stress responses, offers a promising non-invasive, real-time alternative for stress detection. This study examined relationships between speech features, state anger, and salivary cortisol using a validated stress-induction paradigm.

**Methods:**

Participants (*N* = 82) were assigned to cold (*n* = 43) or warm water (*n* = 39) groups. Saliva samples and speech recordings were collected before and 20 minutes after the Socially Evaluated Cold Pressor Test (SECPT), alongside State–Trait Anger Expression Inventory (STAXI) ratings. Acoustic features from frequency, energy, spectral, and temporal domains were analysed. Statistical analyses included Wilcoxon tests, correlations, linear mixed models (LMMs), and machine learning (ML) models, adjusting for covariates.

**Results:**

Post-intervention, the cold group showed significantly higher cortisol and state anger. Stress-related speech changes occurred across domains. Alpha ratio decreased and MFCC3 increased post-stress in the cold group, associated with cortisol and robust to sex and baseline levels. Cortisol–speech correlations were significant in the cold group, including sex-specific patterns. LMMs indicated baseline cortisol influenced feature changes, differing by sex. ML models modestly predicted SECPT group membership (AUC = 0.55) and showed moderate accuracy estimating cortisol and STAXI scores, with mean absolute errors corresponding to ∼ 24–38% and ∼16–28% of observed ranges, respectively.

**Conclusion:**

This study demonstrates the potential of speech features as objective stress markers, revealing associations with cortisol and state anger. Speech analysis may offer a valuable, non-invasive tool for assessing stress responses, with notable sex differences in vocal biomarkers.

## Significant outcomes

- Speech features showed clear differences between cold and warm conditions, reflecting stress-related changes.
- Alpha ratio and MFCC3 emerged as the most reliable speech markers of stress, capturing involuntary physiological vocal changes.
- Local shimmer also indicates stress through reduced amplitude variability, while frequency-based features like jitter and pitch are less reliable due to sex and individual variability.

## Limitations

- Variability in technical equipment across recordings may affect the comparability of acoustic features.
- The study did not control for participants’ circadian rhythms, which can influence cortisol measurements.
- The SECPT stress induction paradigm may not fully represent the complexity of real-world stress responses.
- The study lacks a recovery-phase cortisol measure, limiting interpretation of whether speech changes reflect peak stress or recovery.

## Introduction

Stress is a complex physiological and psychological response to external and internal stimuli. A key biological marker of stress is cortisol, a glucocorticoid hormone released by the adrenal cortex (Grant *et al*., 1957). Cortisol plays a crucial role in stress response, impacting metabolism, immune response, and central nervous system activity (Marieb, 2007). Elevated cortisol levels are associated with heightened stress, making it a reliable biomarker for stress assessment (Dickerson & Kemeny, 2004; Hellhammer *et al*., 2009).

Stress is a significant risk factor for poor health (O’Connor *et al*., 2021), including depression and anxiety disorders (Hammen, 2015; Pêgo *et al*., 2010).Underestimating stress is associated with long-term development of depressive symptoms (Izawa *et al*., 2016). Given these risks, objective and accurate stress measurement is crucial for better management and improved outcomes under pressure (Liu *et al*., 2019). However, traditional methods like self-report questionnaires are subjective and susceptible to bias. This underscores the need for objective, reliable, and non-invasive stress biomarkers (Slavich, 2019).

Multimodal wearable devices studies have shown that combinations of physiological signals (e.g., heart rate variability, electrodermal activity, electroencephalography, blood-volume pulse, inter-beat interval, skin temperature) can distinguish stress states and track changes referenced to salivary cortisol (Betti *et al*., 2018; Nath & Thapliyal, 2021). Another promising measure for stress biomarker development might be speech, as a natural and ubiquitous mode of communication. A direct link between physical state and speech production has been proposed several decades ago (Scherer, 1986). Since stress increases muscle tension and respiration rate, which subsequently affect speech production, research suggests stress could be identifiable through the characteristics of speech (Sondhi *et al*., 2015). These include speech fluency (Buchanan *et al*., 2014), fundamental frequency, i.e. pitch (Rajasekaran *et al*., 1986), jitter, speaking rate, and length and number of pauses (J. H. L. Hansen & Patil, 2007a). Recent findings further highlight stress effects on features like fundamental frequency (F0), Harmonics-to-Noise Ratio (HNR), and shimmer (Kappen *et al*., 2022).

Research shows significant sex differences in voice quality under stress. Women tend to experience more vocal symptoms and stress symptoms than men (Holmqvist *et al*., 2013). Under stress, female voices become breathier, more strained, and exhibit lower fundamental frequency, intensity, and aerodynamic capacity (Van Lierde *et al*., 2009). For high-anxiety females, articulation precision increases under cognitive stress but decreases under emotional stress (Tolkmitt & Scherer, 1986). Stress-induced changes in muscle tension can lead to increased F0 floor in both high-anxious and anxiety-denying individuals (Tolkmitt & Scherer, 1986). These findings suggest stress affects vocal quality differently by sex, with women showing more pronounced changes.

Using speech to detect stress offers advantages like continuous, real-time, and non-invasive monitoring (Robin *et al*., 2020), making it ideal in environments where traditional methods are impractical. In telemedicine and remote patient monitoring settings, speech analysis could provide immediate feedback on patient stress levels without requiring physical presence or complex equipment (König *et al*., 2021).

The link between speech features and cortisol, a validated physiological stress marker, remains underexplored. Pisanski and Sorokowski (2021) found that elevated salivary cortisol levels in 10 female students during a real-life stressful situation correlated with increased pitch, altered vocal tract resonances, and changes in speech speed, leading listeners to perceive these voices as more stressed. Baird and colleagues (2021) used a multi-modal approach and identified significant speech features, including pitch, speech rate, and formant frequencies as effective predictors of physiological stress markers including saliva-based cortisol levels, heart rate, and respiration. Other multimodal studies using biosignals and machine learning have achieved up to 95.21% accuracy in classifying stress states (Aigrain *et al*., 2018; Bobade & Vani, 2020). Despite these findings, gaps remain in understanding how specific speech features correlate with cortisol fluctuations across various contexts and populations.

Combining speech analysis with cortisol measurement offers a promising approach to stress detection, paving the way for precise diagnostic tools. Validating speech-based biomarkers against objective stress indicators like cortisol is essential to ensure their reliability and utility, particularly for early diagnosis of stress-related disorders or real-time monitoring in high-pressure professions. This study investigates the associations between speech features and cortisol levels to evaluate the potential of speech as an objective stress biomarker. Based on existing literature, we posit that features such as increased voice pitch, altered vocal tract resonances, voice quality features, and changes in speech speed will correlate with elevated cortisol levels following the stress-inducing paradigm. Due to inconsistent findings, the direction of these effects remains uncertain.

Our primary aim was to test whether acute stress induced by the Socially Evaluated Cold Pressor Test (SECPT) produces vocal changes that covary with hypothalamic–pituitary–adrenal activation indexed by salivary cortisol reactivity measured 20 minutes after stressor onset. We examined whether a set of vocal features reflects acute stress when anchored to a physiological marker of HPA activation. Specifically, we tested whether changes in selected acoustic parameters from pre- to post-SECPT relate to salivary cortisol reactivity and whether these relationships differ between sexes.

## Material & methods

### Participants

Healthy participants were recruited for this study over a 15-month period at the Department of Psychiatry, University of Frankfurt, Germany. The data reported here stem from two separate experiments within the larger project ‘Identifying Mediators of Stress-Aggression and Experimental Manipulation by tDCS’, approved by the Ethics Committee of the Goethe University Frankfurt Medical Faculty (Ref: 20-1035). The study adhered to the Declaration of Helsinki. All participants provided written informed consent and received €10 per hour for their participation.

Inclusion criteria required participants to be right-handed, aged 18–55, German language proficiency at C1 level, with no confirmed psychiatric, neurological, or cardiovascular conditions. To exclude cognitive impairments and low IQ (<70), we administered the Trail-Making Test (Reitan, 1955) and the Mehrfachwahl-Wortschatz-Intelligenztest (MWT-B, Lehrl, 2005). Additional exclusion criteria included smoking (more than 10 cigarettes/day), caffeine intake above 400 mg/day, adipositas, and any medication with potential effects on the HPA axis activity, e.g., current use of cortisone nasal spray. Participants were instructed to abstain from alcohol and other drugs for 48 hours prior to testing. If any participant showed signs of a clinically significant psychiatric or somatic condition during the assessment, they were advised to seek appropriate clinical care.

#### Study overview

**Figure 1.**
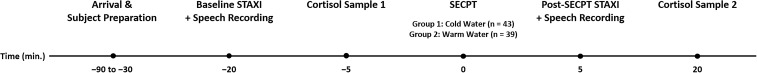
Graphical overview depicting the study timeline.

This study employed a randomised 2 × 2 factorial design with *time point* (pre-stressor vs. post-stressor) as the within-subject factor and *group* (cold water vs. warm water) as the between-subject factor. All procedures were conducted at the Department of Psychiatry, Psychosomatics, and Psychotherapy, Goethe University Hospital Frankfurt, Germany. To control for cortisol’s diurnal variation, all experimental sessions were scheduled between 2:00 PM and 6:00 PM (Lee *et al*., 2015). Fig. 1 provides an overview of the study timeline.

Upon arrival, participants received detailed study information. Subsequently, the experimenter documented recent behaviours affecting cortisol levels, including food intake, smoking, caffeine consumption, gum chewing, tooth brushing, and physical activity (Fukuda & Morimoto, 2001; Pritchard *et al*., 2017) and verified compliance with pre-experimental restrictions. To minimise confounders, subjects were not allowed to eat or drink anything other than water for one hour before the testing session started..

Baseline stress assessments included the first salivary cortisol sample (see cortisol assessment), completion of the State–Trait Anger Expression Inventory (STAXI), and a speech recording (see Psychological assessment and speech recording). Afterwards stress induction was achieved using the Socially Evaluated Cold Pressor Test (SECPT, see Stress test procedure), followed by a second STAXI and speech recording. The second cortisol sample was obtained 20 minutes after SECPT onset. For half the participants, the same procedure was followed with an added EEG recording, not included in this analysis..

### Stress test procedure

To induce stress, we used the Socially Evaluated Cold Pressor Test (SECPT), a well-established paradigm combining physical discomfort and social evaluation to elicit psychosocial stress (Schwabe & Schächinger, 2018). The cold water triggers a physiological stress response by activating nociceptors, sending signals to the hypothalamus, the primary stress regulator. Simultaneous introduces a psychosocial stressor, further amplifying the response. This combination is particularly effective in activating the Hypothalamic-Pituitary-Adrenal (HPA) axis, as shown by elevated salivary cortisol levels.

Participants were randomly assigned to a cold or warm water group. In the cold water group (*N* = 43), participants immersed their right hand up to the wrist in 3–5°C water. They were informed that the session would be video-recorded to study their reactions (Schwabe *et al*., 2008) asked to look into a camera, and instructed to keep their hand submerged for as long as possible, up to 3 minutes. A researcher observed them throughout. No actual filming took place, which was disclosed during debriefing. Participants in the warm water group (*N* = 39) immersed their hands in 35–37°C warm water for 3 minutes without video-recording.

### Psychological assessment and speech recording

To evaluate psychological stress, participants self-reported anger symptoms before and after the intervention using the State–Trait Anger Expression Inventory (STAXI, Spielberger *et al*., 1999). Participants also completed a speech task, reading 20 short sentences (e.g., ‘The rag is on the freezer’. ‘They just carried it upstairs and now they’re going down again’.) both pre- and post-intervention. A full sentence list is provided in the supplementary material. Responses were recorded using different mobile phones and stored in .wav-Format.

### Cortisol assessment

Two saliva samples were collected from all participants in each session: the first after completing the initial questionnaires, and the second 20 minutes after SECPT onset. Saliva was collected using the ‘Salivette’ (Sarstedt AG & Co., Nümbrecht, Germany) which includes a cotton swab suspended in a centrifuge vessel. Participants gently chewed the cotton swab for one minute, then returned it to the insert without touching. Salivary samples were stored at −30°C until the biochemical analysis, which was performed by the Group of Translational Psychiatry, University Hospital Frankfurt, Germany. Samples were stored for a maximum of 47 days until analyses. The concentration of free salivary cortisol was analysed using a luminescence immunoassay (IBL, Hamburg, Germany) with intra- and inter-assay precision of 4.5% and 4.3%, respectively.

### Speech feature processing

Automatised acoustic feature extraction was conducted with our own speech processing library ‘Sigma‘ using Python 3.9. Scripts are available upon reasonable request from the corresponding author.

We investigated several categories of acoustic speech features previously associated with stress symptoms (Buchanan *et al*., 2014; J. Hansen & Patil, 2007a; Sondhi *et al*., 2015). Attributes were categorised into groups (refer to Supplementary Table 1 for groupings and corresponding attributes), including frequency, energy, spectral and temporal features. Frequency features consist of variables associated with the formants F0 to F3. F0 is perceived as voice pitch (Ladefoged, 1996), while formants F1-F3 contribute to vowel sound articulation (Ladefoged & Johnson, 2011). Energy features included jitter(voice instability), shimmer (variability in loudness or intensity), and the harmonics-to-noise ratio (HNR (Teixeira *et al*., 2013)). Spectral features describe frequency characteristics, such as the mel frequency cepstral coefficient (MFCC), often used in speaker identification (Nakagawa *et al*., 2007). Additionally, we employed features defined by our research team concerning the temporal dimensions of speech (König *et al*., 2019), capturing timing aspects of speech, such as duration, rhythm, and temporal patterns (Zellner, 1994). In total 65 features were extracted; 10 energy-related features, 23 frequency-related features, 22 spectral features, and 10 temporal features.

### Statistical analysis

#### Power analysis

The required sample size was determined using G*Power(Faul *et al*., 2007) with *α* = 0.05 (one-tailed), *β* = .80. A small (.25) effect size with two groups (cold/warm water) and two measurements (pre/post measurement) in a repeated measure ANOVA with within and between factor interaction was used in order to calculate the sample size. A correlation of 0.57 between repeated measures, based on the internal consistency of the STAXI-II subscales (Schamborg *et al*., 2016) informed the calculation. To allow for separate analysis by sex, a total of *N* = 60 participants was required.

#### Descriptive statistics

Statistical analyses were conducted with the Python package scipy.stats (v1.11.4, Linux v5.10.0). Descriptive statistics were computed for demographic variables, cortisol, and STAXI values.

#### Speech features

Within-group differences in speech features pre- and post-intervention were assessed using the paired Wilcoxon test, a non-parametric method suitable for small samples or non-normal data (Wilcoxon, 1945).

Each of the twenty sentences was analysed independently. Sentence order effects were controlled by including sentence ID as a covariate. *P* values were adjusted for multiple hypothesis testing using the Benjamini–Hochberg procedure (Benjamini & Hochberg, 1995) clustered in categories (see Supplementary Table 1).

To assess associations between speech features and cortisol/STAXI values while accounting for baseline differences, we computed changes scores (post–pre) for each measure. Spearman correlations were then calculated between these difference scores. Sex-based differences in correlation strength were assessed using Fisher’s *Z* transformation to compare correlation coefficients between male and female participants.

Linear models (LMM) were applied to examine the relationship between cortisol changes and speech feature changes, accounting for potential moderating effects of group, sex, and baseline cortisol levels. The model used was: Cortisol_Change ∼ Group * Feature_Change + Sex + Cortisol_Before. LMMs accounted for repeated measures by including random effects for participant-specific intercepts and slopes, while fixed effects modelled relationships between speech features and cortisol change. Outliers were removed using the interquartile range (IQR) method (Vinutha *et al*., 2018), applied independently by sex and experimental group for each speech feature. All change scores were calculated as post-intervention minus pre-intervention values.

To evaluate the predictive value of speech features, machine learning models were trained to (1) classify group membership (cold vs. warm) and (2) predict cortisol levels and STAXI scores. To control sex-related variability, each acoustic feature was regressed on sex, and the residuals were used as sex-normalised inputs. All features were *Z*-score standardised. Models were trained on the difference scores (after - before) to capture intervention effects. Five classifier algorithms were evaluated: support vector machines (SVM), extra trees (XT), random forests (RF), linear models (LM), and decision trees (DT). Model performance was assessed using 10-fold group cross-validations, with folds stratified by participant ID to prevent data leakage. Stepwise feature selection was applied: models were iteratively trained using an increasing number of top-ranked features to determine the optimal set. Classification performance was assessed using the area under the ROC curve (AUC), while regression performance was evaluated using mean absolute error (MAE).

## Results

A total of 82 participants were included, each undergoing repeated measures at two time points: before and after exposure to the experimental conditions (cold/warm water). Table 1 demonstrates STAXI scores and cortisol levels for both groups (cold/warm) and sexes.

**Table 1.** Demographics, STAXI scores and cortisol data

				STAXI scores			Cortisol Level		
Condition	Sex	*N*	Age	Before	After	*p*	Before	After	*p*
Cold	All	43	23.67(5.36)	15.84(1.49)	17.28(3.13)	<0.001	4.18(3.28)	8.76(7.35)	<0.001
	Female	23	24.30(6.72)	15.78(1.65)	17.61(3.81)	<0.001	3.53(2.88)	6.88(3.70)	<0.001
	Male	20	22.95(3.19)	15.90(1.33)	16.90(2.13)	<0.001	4.92(3.61)	10.92(9.71)	<0.001
Warm	All	39	23.95(5.47)	15.82(1.35)	15.33(0.90)	<0.001	4.74(4.49)	3.48(2.80)	<0.001
	Female	18	22.72(3.43)	15.56(0.92)	15.22(0.94)	<0.001	3.74(2.29)	2.93(1.81)	<0.001
	Male	21	25.00(6.66)	16.05(1.63)	15.43(0.87)	<0.001	5.60(5.67)	3.95(3.41)	<0.001

There were no statistically significant differences in STAXI scores between sexes at either time point, based on Wilcoxon rank-sum test (*t_0_*: *W* = 730, *p* = 0.247; *t_0_*: *W* = 802, *p* = 0.693). Similarly, Cortisol levels did not differ significantly between sexes (*t_0_*: *W* = 693, *p* = 0.174; *t_0_*: *W* = 738, *p* = 0.346).

Age also did not differ significantly between cold and warm water groups (*W* = 851, *p* = 0.911), suggesting that demographic and baseline characteristics were comparable across the conditions and sexes.

Cortisol and STAXI state of anger levels changed significantly pre- to post intervention. In the cold water condition, participants exhibited a significant increase in cortisol (*W* = 58,100, *p* < 0.001) and STAXI scores (*W* = 23,390, *p* < 0.001) after SECPT. Conversely, participants in the warm water condition showed a significant decrease in cortisol (*W* = 26,680, *p* < 0.001) and STAXI scores (*W* = 7,580, *p* < 0.001).

When analysed by sex, similar patterns emerged. Among females, cortisol (*W* = 6,630, *p* < 0.001) and STAXI scores (*W* = 1,590, *p* < 0.001) significantly decreased after warm water exposure, whereas both cortisol (*W* = 1,787, *p* < 0.001) and STAXI scores (*W* = 5,430, *p* < 0.001) significantly increased after cold water exposure. Among males, cortisol (*W* = 6,630, *p* < 0.001) and STAXI scores (*W* = 107.8, *p* < 0.001) significantly decreased after exposure to the warm water condition. In contrast, cortisol (*W* = 11,850, *p* < 0.001) and STAXI scores (*W* = 6,390, *p* < 0.001) significantly increased after cold water exposure.

### Differences in Speech Features before and after Intervention

A Wilcoxon test was conducted to compare pre- and post SECPT timepoints for both conditions (cold and warm, see Table 2). Reported *p* values reflect these comparisons..

**Table 2.** Difference in speech features before and after intervention. Features that remained significant after correction are highlighted in bold. Before and after arrows indicate if the feature value increased or decreased in value after exposure to cold/warm water. Features are organised by feature type and then alphabetically. Only features that were significant in the warm and/or cold group are summarised in Table 2. All results are listed in Supplementary Table 2

Feature Type	Feature	WARM			COLD		
		**Effect Size**	**Before v. After**	***p*(adj)**	**Effect Size**	**Before v. After**	***p*(adj)**
Energy	**hnr_mean**	**−0.229**	**<**	**<0.001**	−0.067	<	0.102
	**hnr_sd**	**−0.244**	**>**	**<0.001**	−0.07	>	0.091
	**loudness_mean**	−0.077	>	0.071	**−0.128**	**<**	**0.001**
	**loudness_sd**	**−0.131**	**>**	**0.001**	−0.057	<	0.17
	**rate_loudness_peaks**	**−0.112**	**<**	**0.009**	**−0.159**	**<**	**<0.001**
Frequency	**f1_bandwidth_mean**	**−0.167**	**<**	**<0.001**	−0.009	>	0.957
	**f1_bandwidth_sd**	**−0.118**	**<**	**0.003**	−0.06	>	0.158
	**f2_frequency_mean**	**−0.225**	**<**	**0.00**	−0.07	>	0.091
	**f2_frequency_sd**	−0.041	<	0.36	**−0.085**	**>**	**0.047**
	**f3_bandwidth_mean**	−0.048	>	0.275	**−0.218**	**<**	**<0.001**
	**f3_frequency_mean**	**−0.169**	**<**	**<0.001**	−0.081	<	0.059
	**pitch_mean**	**−0.115**	**>**	**0.004**	−0.033	<	0.477
	**pitch_min**	**−0.083**	**>**	**0.05**	−0.017	>	0.796
	**pitch_std**	**−0.16**	**<**	**<0.001**	−0.069	<	0.094
Spectral	**average_mfccs_1**	**−0.254**	**>**	**<0.001**	−0.001	<	0.986
	**average_mfccs_2**	**−0.136**	**<**	**0.001**	**−0.088**	**<**	**0.038**
	**average_mfccs_3**	**−0.096**	**<**	**0.018**	**−0.229**	**>**	**<0.001**
	**average_mfccs_4**	−0.063	>	0.159	**−0.131**	**>**	**0.001**
	**f1_relative_energy_mean**	**−0.129**	**>**	**0.001**	−0.004	>	0.986
	**f3_relative_energy_mean**	−0.058	<	0.193	**−0.17**	**>**	**<0.001**
	**h1_a3_harmonic_difference_mean**	**−0.13**	**<**	**0.001**	**−0.162**	**>**	**<0.001**
	**h1_a3_harmonic_difference_sd**	−0.001	>	0.967	**−0.117**	**>**	**0.003**
	**h1_h2_harmonic_difference_mean**	−0.02	>	0.661	**−0.09**	**>**	**0.035**
	**h1_h2_harmonic_difference_sd**	**−0.135**	**>**	**0.001**	**−0.145**	**>**	**<0.001**
	**hammarberg_index_mean**	**−0.187**	**>**	**<0.001**	−0.073	<	0.083
	**spectral_slope_0_500_mean**	**−0.122**	**>**	**0.003**	−0.007	>	0.957
	**spectral_slope_0_500_sd**	−0.059	>	0.19	**−0.125**	**>**	**0.001**
	**spectral_slope_500_1500_mean**	**−0.102**	**>**	**0.012**	−0.054	>	0.18
Temporal	**duration**	**−0.414**	**>**	**<0.001**	**−0.383**	**>**	**<0.001**
	**number_of_pauses**	−0.33	>	0.106	**−0.428**	**>**	**<0.001**
	**pause_durations_mean**	−0.12	>	0.003	−0.063	>	0.165
	**pause_durations_sd**	**−0.106**	**>**	**0.009**	**−0.119**	**>**	**0.002**
	**pause_durations_sum**	**−0.189**	**>**	**<0.001**	**−0.193**	**>**	**<0.001**
	**speech_ratio**	**−0.097**	**<**	**0.017**	−0.002	<	0.986
	**utterance_durations_mean**	**−0.125**	**>**	**0.003**	−0.078	<	0.083
	**utterance_durations_sum**	**−0.241**	**>**	**<0.001**	**−0.26**	**>**	**<0.001**

**Energy** features revealed distinct patterns between groups. Loudness rate increased in both groups. However, while mean loudness decreased in the warm group (n.s.), it exhibited a significant increase in the cold group. The mean harmonic-to-noise ratio (HNR) showed a significant increase in the warm group, accompanied by a decrease in HNR standard deviation. The cold group showed similar trends, though not statistically significant.

**Frequency** features revealed opposing trends. In the warm group, both the mean and standard deviation (SD) of F1 bandwidth significantly increased, while both decreased in the cold group (n.s.). The mean F2 frequency significantly increased in the warm group and decreased (n.s.) in the cold group. Conversely, the SD of F2 frequency significantly decreased in the cold group, and increased (n.s.) in the warm group. The mean F3 frequency increased in the warm group, and F3 bandwidth increased in the cold group. Mean pitch significantly decreased in the warm group, whereas the cold group exhibited a non-significant opposite trend. Pitch minimum decreased and pitch SD increased in the warm group, with the same pattern observed in the cold group but without statistical significance. Overall, formant features displayed opposing trends, but were rarely significant in both groups for the same features.

The analysis of **spectral** features further supported group-specific trends. Mel-frequency cepstral coefficients (MFCC) 1, significantly decreased in the warm group but showed an non-significant increase in the cold group. MFCC2 significantly decreased in both groups. MFCC3 exhibited significant opposing patterns, increasing in the warm group and decreasing in the cold group. MFCC4 significantly decreased in the cold group, with the same pattern observed in the warm group but without statistical significance. Regarding relative mean energy, F1 relative mean energy significantly decreased in the warm group and showed a non-significant decrease in the cold group. F3 relative mean energy significantly decreased in the cold group, whereas the warm group displayed an opposite but non-significant trend. The mean H1_a3 difference significantly increased in the warm group but significantly decreased in the cold group. Additionally, the H1_a3 difference SD and the mean H1_H2 difference significantly decreased in the cold group, with the same non-significant patterns observed in the warm group. The harmonic difference H1_H2 SD significantly decreased in both groups. The Hammarberg index significantly decreased in the warm group, whereas the cold group exhibited a non-significant increase. The spectral slope within the 0–500 Hz and 500–1500 Hz ranges decreased in both the warm and cold groups.

Finally, the analysis of **temporal** features indicated significant changes after exposure across several measures. Duration, pause duration, and the total utterance time significantly decreased following exposure. Notably, all but one (mean utterance duration) of the temporal features exhibited the same pattern in both the warm and cold groups.

### Speech feature correlations with cortisol levels and STAXI scores

#### Cortisol levels

**Figure 2.**
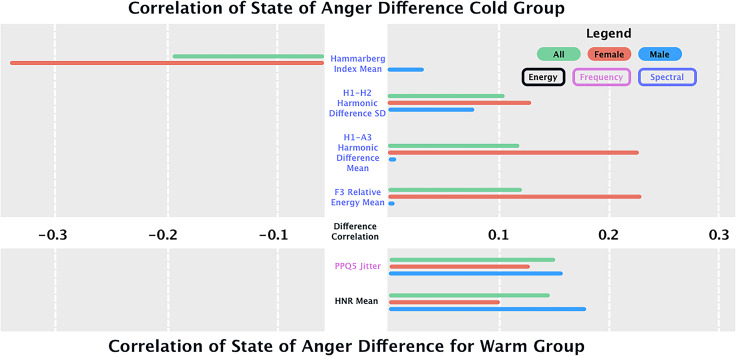
Delta in correlations of speech features and cortisol differences (after-before) for cold and warm groups for the whole sample and stratified by sex. For brevity reasons, only significant results are displayed here, the full results are displayed in Supplementary Table 3.

Spearman correlations between each feature and cortisol levels (Table 3) and STAXI scores (Supplementary Table 4) were calculated using the after-before difference. Only significant results are presented in Fig. 2 and Table 3, with full results in Supplementary Table 3.

**Table 3.** Associations of acoustic features with cortisol levels across conditions. The table presents correlation coefficients (corr), effect sizes (ES), and adjusted *p*-values (*p*-adj) for the overall population. Sex-specific correlations are indicated separately for males (M) and females (F), with positive ( + ) or negative ( − ) directions. Fisher’s *Z*-test results, including *Z*-values and corresponding *p*-values, are reported under the ‘Sex Comparison’ header to assess differences between sexes. Results are shown for both cold and warm conditions. For brevity reasons, only significant results are reported here. The full results are displayed in Supplementary Table 3

Cortisol Levels Difference Exposure to Stress Condition		COLD							WARM						
		***Overall***			***Sex Comparison***				***Overall***			***Sex Comparison***			
**Feature Type**	**Feature**	**Corr**	**ES**	***p*-adj**	**M**	**F**	***Z***	***p***	**Corr**	**ES**	***p*-adj**	**M**	**F**	***Z***	***p***
**Energy**	loudness_mean	−0.18	0.36	<0.001	–	–	−0.18	0.86	−0.01	0.02	0.91	–	+	−0.18	0.86
**Frequency**	ddp_jitter	−0.1	0.2	0.03	–	–	−0.19	0.85	0.06	0.13	0.3	+	+	−0.19	0.85
	local_absolute_jitter	−0.1	0.21	0.02	–	–	−0.97	0.33	0.08	0.15	0.3	+	–	−0.97	0.33
	ppq5_jitter	−0.1	0.2	0.02	–	–	−0.46	0.65	0.1	0.2	0.08	+	+	−0.46	0.65
	rap_jitter	−0.1	0.2	0.03	–	–	−0.19	0.85	0.06	0.13	0.3	+	+	−0.19	0.85
**Spectral**	alpha_ratio_mean	−0.12	0.24	0.01	–	–	0.3	0.77	−0.03	0.06	0.77	–	+	0.3	0.77
	average_mfccs_1	−0.16	0.31	<0.001	–	–	1	0.32	0.1	0.2	0.09	+	+	1	0.32
	average_mfccs_2	0.03	0.07	0.57	+	+	0.39	0.69	−0.16	0.32	<0.001	–	–	0.39	0.69
	average_mfccs_4	0.09	0.19	0.03	–	+	−2.68	0.01	−0.07	0.13	0.3	–	–	−2.68	0.01
	f1_relative_energy_sd	−0.1	0.2	0.02	–	–	0.63	0.53	−0.06	0.13	0.3	–	–	0.63	0.53
	f3_relative_energy_mean	0.11	0.23	0.01	+	+	−1.22	0.22	0	0	1	+	–	−1.22	0.22
	hammarberg_index_mean	−0.12	0.24	0.01	–	–	2.89	<0.001	0.02	0.03	0.9	–	+	2.89	<0.001

For **energy** features, mean loudness was significantly correlated with cortisol levels in the cold condition (*r* = −0.18, *p* <0.001), while the warm condition showed a weaker and non-significant correlation (*r* = −0.01, *p* = 0.91).

Within **frequency** features, all jitter measures (DDP jitter (*r* = −0.10, *p* = 0.03), local absolute jitter (*r* = −0.10, *p* = 0.02), PPQ5 jitter (*r* = −0.10, *p* = 0.02), and RAP jitter (*r* = −0.10, *p* = 0.03) were significantly negatively correlated with cortisol levels in the cold condition. These relationships were weaker and non-significant in the warm group (*p* >0.08).

For **spectral** features, the mean alpha ratio showed a small but significant negative correlation with cortisol levels in the cold condition (*r* = −0.12, *p* = 0.01), while no notable association was found in the warm condition (*r* = −0.03, *p* = 0.77). MFCC1 was negatively correlated with cortisol in the cold condition (*r* = −0.16, *p*<0.001) but not significantly associated with the warm condition (*r* = 0.10, *p* = 0.09). In contrast, MFCC2 exhibited a significant negative correlation in the warm condition (*r* = −0.16, *p* <0.001) but not in the cold condition (*r* = 0.03, *p* = 0.57). MFCC4 displayed opposite patterns across conditions, with a weak positive correlation in the cold condition (*r* = 0.09, *p* = 0.03) and a weak negative correlation in the warm condition (*r* = −0.07, *p* = 0.3), with a significant sex difference (*Z* = −2.68, *p* = 0.01).

Regarding formant energy measures, F1 relative energy SD showed a significant negative correlation with cortisol in the cold condition (*r* = −0.10, *p* = 0.02), but not in the warm group (*r* = −0.06, *p* = 0.3). Mean F3 relative energy was positively correlated with cortisol in the cold condition (*r* = 0.11, *p* = 0.01) but not in the warm condition (*r* = 0.0, *p* = 1.0).

The Hammarberg index demonstrated a significant negative correlation with cortisol levels in the cold condition (*r* = −0.12, *p* = 0.01), with a strong sex effect (*Z* = 2.89, *p* <0.001). In the warm condition, this relationship was weak and not significant (*r* = 0.02, *p* = 0.9).

#### STAXI scores

**Figure 3.**
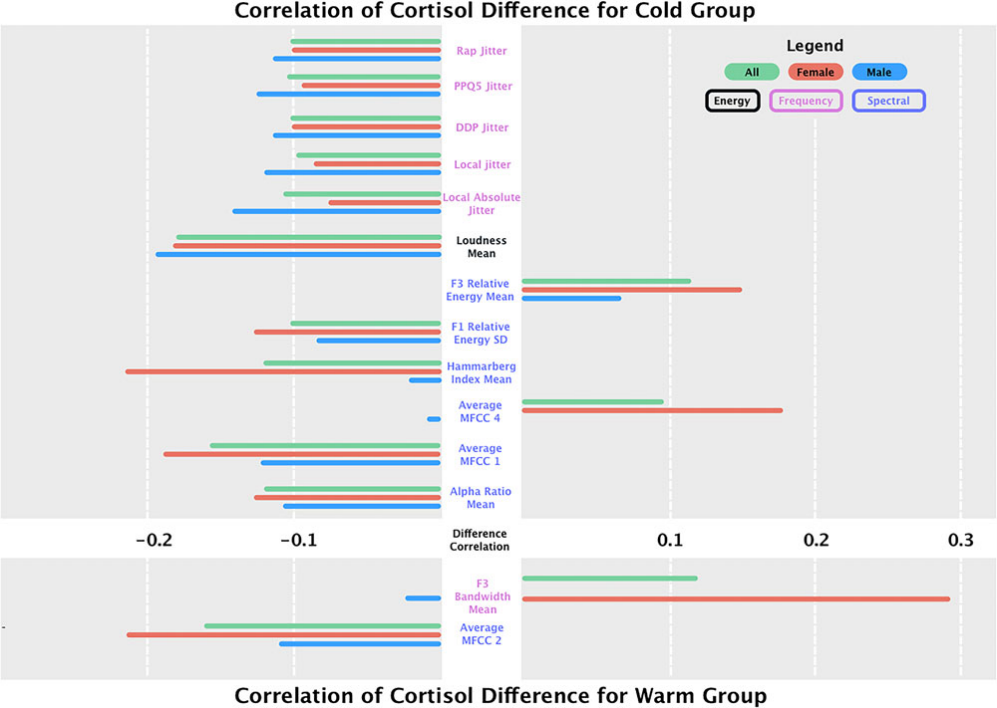
Delta in correlations of speech features and STAXI scores (after-before) for cold and warm groups for the whole sample and stratified by sex. For brevity reasons, only significant results are displayed here, the full results are displayed in Supplementary Table 4.

The relationship between acoustic features and STAXI scores varied across the cold and warm stress conditions. Only significant results are presented in Fig. 3, full results are displayed in Supplementary Table 4.

For **energy** features, HNR was negatively but not significantly correlated with STAXI scores in the cold condition (*r* = −0.09, *p* = 0.08), while in the warm condition, it was significantly positively correlated (*r* = 0.14, *p* < 0.001). No significant sex differences were observed in either condition.

Regarding **frequency** features, PPQ5 jitter was not significantly correlated with STAXI scores in the cold condition (*r* = −0.04, *p* = 0.48). However, in the warm condition, it showed a significant positive correlation (*r* = 0.14, *p* < 0.001). No significant sex differences were found.

For **spectral** features, the SD of the H1-H2 harmonic difference was significantly positively correlated with STAXI scores in the cold condition (*r* = 0.10, *p* = 0.05), but not in the warm condition (*r* = 0.02, *p* = 0.81), with no significant sex differences. The Hammarberg index was significantly negatively correlated with STAXI scores in the cold condition (*r* = −0.13, *p* < 0.001), with a significant sex difference (*Z* = 4.62, *p* < 0.001). In the warm condition, the correlation was positive but not significant (*r* = 0.09, *p* = 0.12), with no significant sex effect.

#### Linear mixed model analyses

Linear mixed model analyses examining the relationships between cortisol changes, baseline cortisol levels, sex, and changes in speech features revealed several significant findings. Higher baseline cortisol levels were linked to changes in average_mfccs_3 (*β* = −0.61, *p* = 0.014) and local_shimmer (*β* = −0.20, *p* = 0.003). Changes in cortisol (after - before) were negatively associated with changes in mean alpha ratio (*β* = −0.05, *p* = 0.02), indicating that larger cortisol changes were linked to smaller changes in this feature.

Sex differences were also observed: male participants exhibited consistently negative associations with changes in multiple speech features, including features mirroring shimmer, jitter, and pitch. Supplementary Table 5 displays the detailed results.

#### Machine learning model performance

The highest AUC was achieved by the SVM classifier using difference-based features (AUC = 0.55, *k* = 15), followed closely by XT (AUC = 0.54) and RF (AUC = 0.53). For regression tasks, SVMs also yielded the best performance in predicting both cortisol and state anger scores across difference-based input. MAEs for cortisol prediction ranged from approximately 3.8 to 6.0 nmol/L, while those for STAXI scores ranged from ∼ 1.3 to 2.2. Full model metrics, including performance across all time points and model types, are presented in Supplementary Table 6. The most frequently selected features across tasks were related to spectral shape and shimmer variability, particularly MFCC 2 and 3s, and voice quality metrics (e.g., apq5_shimmer, f1_bandwidth_mean). Supplementary Table 7 presents the ten most frequently selected features across models.

To support interpretation, a summary table was created (Table 4), consolidating key findings across different analyses. It highlights features significantly affected by sex, linked to stress responses, and robust to potential confounders.

**Table 4.** Summary of speech feature associations with sex, stress, and robustness to confounding factors. The table categorises key speech features based on their sensitivity to sex differences (✔ = significant, ✘ = not significant), their link to stress responses (as measured by cortisol levels), and whether these associations are robust to confounding factors such as baseline cortisol and sex. The significant Before v. After column indicates whether a feature showed significant differences before and after exposure to the stress condition and if so, in which condition. Cortisol correlation direction shows whether the feature has a positive ( + ) or negative ( − ) correlation with cortisol levels. Direction (before-after stress) indicates whether the feature difference increased (↑) or decreased (↓) after exposure to the stress condition

Feature Group	Key Features	Affected by Sex	Linked to Cortisol	Robust to Confounders	Analysis Type	Significant Before v. After	Cortisol Correlation Direction	Direction (Before-After Stress)
**Energy**	apq3_shimmer, dda_shimmer	✔	✘	✘	Linear Model	Warm	Negative	**↓**
	hnr_mean	✘	✘	✘	Correlation	None	Negative	**↓**
	local_shimmer	✔	✔	✘	Linear Model	Warm	Negative	**↓**
	loudness_mean	✘	✘	✘	Correlation	Cold	Negative	**↑**
**frequency**	ddp_jitter, local_jitter,	✔	✘	✘	Linear Model	Cold	Negative	**↓**
	rap_jitter	✔	✔	✘	Linear Model	Cold	Negative	**↓**
	pitch_std	✔	✘	✘	Linear Model	Warm	Negative	**↑**
	pitch_min	✘	✘	✘	Correlation	None	Negative	**↑**
	pitch_mean	✘	✘	✘	Correlation	Warm	Negative	**↑**
**Spectral**	**alpha_ratio_mean**	✘	✔	✔	Linear Model	Cold	Negative	**↓**
	average_mfccs_1, average_mfccs_2	✘	✘	✘	Linear Model	Both	Negative	**↑**
	**average_mfccs_3**	✘	✔	✔	Linear Model	Both	Negative	**↑**
	spectral_slope_0_500, spectral_slope_500_1500	✘	✘	✘	Correlation	Warm	Negative	**↓**

## Discussion

This study explored whether acoustic speech changes could serve as objective stress markers by linking them to cortisol levels and self-reported anger under induced stress. The SECPT effectively elicited a stress response, evidenced by significant increases in cortisol levels and STAXI state anger scores in the cold water condition. In contrast, the warm water condition produced significant decreases in both measures. These findings align with previous research (Schwabe & Schächinger, 2018; Hellhammer *et al*., 2009) demonstrating the sensitivity of cortisol and anger ratings to acute stressors. Speech features varied distinctly between cold and warm conditions, with spectral features demonstrating the greatest potential as stress markers due to their robustness against confounding variables. This suggests that HPA axis reactivity may influence vocal spectral properties. These findings support existing evidence that stress affects speech production through e autonomic nervous system activation, increasing laryngeal muscle tension and altering respiratory patterns, which, in turn, alter speech energy, frequency, and spectral characteristics (Giddens *et al*., 2013).

Voice changes under stress stem from a combination of physical and physiological mechanisms, both voluntary and involuntary. However, not all speech features respond uniformly. Individual variability, such as sex and baseline cortisol, complicates interpretation, emphasising the need for robust, stable acoustic indicators. (Hansen & Patil, 2007a).

Dahl & Stepp (2023) identified spectral and energy-based measures as more reliable than frequency-based features due to greater consistency across sexes. We identified alpha ratio and MFCC3 as especially reliable stress features, capturing involuntary physiological changes linked to laryngeal tension and vocal tract resonance, which are less subject to individual variability. Local shimmer, an energy-based feature, serves as a secondary stress marker, reflecting stress-induced reductions in amplitude variability. In contrast, frequency-based features such as jitter and pitch were more affected by sex and individual differences, limiting their reliability for universal stress detection.

### Influence of baseline cortisol and sex on speech changes

Baseline speech features provide a crucial reference for evaluating stress-induced vocal changes. Notably, baseline cortisol levels significantly influenced changes in MFCC3 (*β* = −0.606, *p* = 0.014) and local shimmer (*β* = −0.201, *p* = 0.003), suggesting that individuals with higher baseline cortisol exhibited greater reductions in these features under stress, a pattern previously reported (Carrillo-González *et al*., 2025). This may reflect reduced vocal flexibility under acute stress, consistent with evidence that elevated baseline cortisol is linked to blunted HPA axis reactivity due to possible desensitisation.

Sex differences also influenced vocal response to stress. Males exhibited lower changes in jitter, shimmer, and pitch-related metrics compared to females, likely due to sex-related differences in neuromuscular control and vocal fold mass. Females generally exhibit more pronounced changes (König *et al*., 2021), possibly due to more dynamic neuromuscular control over vocal functions and smaller vocal folds, which may lead to greater laryngeal tension and pitch variability. In contrast, males’ thicker vocal folds (Zhang, 2021) may provide greater vocal stability under stress.

### State of anger and speech-based stress analysis

Anger affects speech in ways that overlap with, but also differ from, stress-induced vocal changes. In this study, state anger increased significantly in the cold condition but not in the warm condition, suggesting a context-dependent emotional response. Anger was associated with a spectral shift toward lower frequencies, potentially as a compensatory mechanism to enhance vocal dominance. The Hammarberg Index showed a significant negative correlation with STAXI scores in the cold condition, with males exhibiting more pronounced changes than females. These results highlight the need to differentiate anger from stress in speech analysis, as anger uniquely influences vocal intensity and modulation, potentially confounding models designed to detect stress.

### Spectral features

Spectral features, which describe how vocal energy is distributed across frequency bands, appear to be the most stable and effective indicators of stress. Alpha ratio and MFCC3 emerged as the most reliable stress biomarkers. Alpha ratio reflects reduced spectral tilt under stress, indicating increased laryngeal tension and reduced high-frequency harmonics, while MFCC3 captures mid-frequency spectral shifts related to stress-induced muscle tension and breath control (Zhang, 2024). These features are particularly robust due to their resistance to voluntary modulation and individual differences, making them suitable for objective stress detection.

A negative correlation between the Hammarberg index and both cortisol levels and STAXI scores in the cold condition suggests stress is associated with a spectral shift toward lower frequencies. This supports prior findings indicating that stress reduces higher frequency energy, possibly due to increased laryngeal tension or altered vocal fold vibration (Bachorowski & Owren, 1995; Laukkanen *et al*., 2008). The observed sex effects align with research showing males and females exhibit distinct phonatory adjustments under stress, likely due to laryngeal anatomy and hormone-mediated vocal control (Bouhuys *et al*., 1995; Fitch & Giedd, 1999). Although anger has been linked to increased vocal intensity and pitch modulation (Scherer, 1986; Banse & Scherer, 1996), our findings indicate that in stress conditions, anger may coincide with a downward spectral energy shift, possibly reflecting a compensatory mechanism or an adaptive response aimed at enhancing vocal dominance (Briefer, 2012).

### Energy features: amplitude stability as a robust stress marker

Energy features, which capture variations in speech loudness, amplitude stability, and harmonic content, also provide useful stress indicators. Local shimmer showed a significant decrease under stress (*β* = −0.201, *p* = 0.003), indicating that increased laryngeal stiffness stabilises vocal fold vibrations, reducing amplitude variability (Bodaghi *et al*., 2025; Carrillo-González *et al*., 2025; Giddens *et al*., 2013; Zhang, 2024). The observed increase of mean loudness under stress exposure in part contrasts with previous findings. Dietrich and Verdolini Abbott (2012) reported lower vocal intensity during stress, while Huttunen *et al*. (2011) and Mattei *et al*. (2019) found that cognitive load and vocal constraints increased both fundamental frequency and vocal intensity. This discrepancy may arise from the differing study setups. Notably, Dietrich and Verdolini Abbott (2012) only included female participants in their study.

### Frequency features: less reliable due to sex and individual variability

Frequency-based measures such as pitch variability and jitter are commonly associated with stress but were found to be less reliable due to significant sex effects and speaker variability. Among them, rap jitter showed some association with stress, but results were inconsistent across individuals. Additionally, pitch mean and pitch min/max were not significantly correlated with cortisol, suggesting these measures are more influenced by voluntary vocal modulation than physiological stress. Our findings align with previous research demonstrating that jitter and other frequency-based measures are influenced by sex (Brockmann *et al*., 2011). The same study suggests voice sound pressure level (SPL) as a key driver of jitter and shimmer, indicating that the observed sex differences in our study may be partially attributable to variations in voice SPL rather than intrinsic sex effects alone. Additionally, we observed that F2 mean frequency significantly decreased after warm exposure, suggesting a more relaxed vocal tone under low stress. Conversely, F2 frequency SD increased after cold exposure, reflecting greater vocal variability under stress (Protopapas & Lieberman, 1997). These results support the idea that stress-related physiological changes disrupt vocal stability.

### Linear mixed model

Results from our LMM suggest that baseline cortisol and sex are important predictors of changes in specific speech features, with males showing consistently lower changes across jitter, shimmer, and pitch-related metrics, and baseline cortisol levels influencing shimmer and MFCCs. Additionally, changes in cortisol were associated with reduced alpha ratio values, highlighting the physiological impact of cortisol on speech characteristics.

### Machine learning results

Classification performance was modest (max AUC = 0.55), suggesting limited discriminability between SECPT groups based on read speech. This likely reflects low expressive variability in structured speech and subtle group-level effects. In contrast, regression models, especially SVMs, performed better. Cortisol MAEs between 3.8–6.0 nmol/L represented ∼ 24–38% of the observed range, STAXI MAEs between 1.3–2.2 points, accounting for ∼ 16–28% of the observed score range. While group-level classification is challenging, acoustic features moderately captured individual differences in stress responses. The most informative features included spectral and temporal voice characteristics such as MFCCs, shimmer, F1 bandwidth and frequency, duration, and alpha ratio. These features reflect articulatory dynamics, phonatory control, and resonance changes, mechanisms plausibly influenced by physiological and emotional stress (Schewski *et al*., 2025). Their consistent relevance across tasks highlights the potential of acoustic voice metrics as individual-level stress indicators, even in constrained speech contexts.

### Limitations

There are limitations to our study. Recording equipment varied, which may limit comparability of acoustic features across devices. However, it has been demonstrated that different technical devices and recording circumstances have negligible effect on acoustic measures of voice (Awan *et al.*, 2024; van der Woerd *et al*., 2020). Furthermore, we did not account for participants’ circadian rhythm when assessing cortisol levels, which can influence the results (Stalder *et al*., 2016). However, all participants were examined in the afternoon, minimising potential confounding effects associated with the cortisol awakening response. Furthermore the time interval between participants’ arrival and the onset of the stressor was not standardised but varied across individuals in the current study, which may have affected baseline cortisol levels at t0. Future research should aim to control and minimise the latency between arrival and the beginning of the experimental procedure to ensure greater comparability across participants. Although the SECPT is a well-established stress induction paradigm, it may not capture the full complexity of stress responses in real-world situations. Lastly, while the 20-minute post-SECPT sample aligns with standard protocols for capturing peak cortisol response, it does not allow us to determine whether speech changes reflect peak stress or early recovery. Future work should include additional time points to better capture the temporal relationship between speech and physiological stress markers.

## Conclusion

Our results converge with prior research identifying increased voice pitch and altered spectral properties as markers of stress (Paulmann *et al.*, 2016; Kappen *et al*., 2022). Our study extends this work by simultaneously accounting for both physiological and psychological stress markers by exploring a broader range of acoustic features. In doing so, we provide a more comprehensive picture of how stress modulates vocal characteristics. Differences between the cold and warm water groups confirm that stress-induced vocal changes are not only measurable but also systematically linked to physiological stress. Speech analysis thus holds promise as a non-invasive, real-time tool for stress assessment, offering advantages over self-report measures or invasive lab-based methods. Features such as jitter, shimmer, and spectral shifts showed sensitivity to stress-related fluctuations, making them ideal for continuous monitoring. Importantly, speech reflects dynamic physiological states and could aid in detecting both acute and chronic stress, conditions closely tied to mental health. Voice-based markers may also support early detection and management of stress-related disorders like anxiety and depression (Tafet & Nemeroff, 2016; Daviu *et al*., 2019) (Gaikwad & Venkatesan, 2024). By correlating speech patterns with cortisol, clinicians gain real-time insight into stress, enabling earlier and more objective interventions. Not all acoustic features are equally reliable for stress detection, underscoring the importance of selecting robust and objective acoustic features for stress detection.

Future analyses should consider replicating existing findings in larger, more diverse populations and across different stress-inducing contexts. Importantly, since our current approach relies on pre/post comparisons relative to a defined baseline, future work should explore how speech-based stress detection can be adapted for real-world settings where baseline definitions are less straightforward. This could involve passive data collection to estimate individual baselines or models that operate without requiring baseline data. Additionally, integrating speech analysis with other physiological measures and exploring its use in clinical practice could further refine its potential for real-time stress monitoring.

## Supporting information

Menne et al. supplementary material Menne et al. supplementary material

## Supplementary material

The supplementary material for this article can be found at https://doi.org/10.1017/neu.2025.10037.

## References

[ref1] Aigrain J , Spodenkiewicz M , Dubuisson S , Detyniecki M , Cohen D and Chetouani M (2018) Multimodal stress detection from multiple assessments. IEEE Transactions on Affective Computing 9(4), 491–506. DOI: 10.1109/TAFFC.2016.2631594 IEEE Transactions on Affective Computing.

[ref2] Awan SN , Bahr R , Watts S , Boyer M , Budinsky R and Bridge2AI Voice Consortium, Bensoussan Y. (2024) Validity of acoustic measures obtained using various recording methods including smartphones with and without headset microphones. Journal of Speech, Language, and Hearing Research: JSLHR 67(6), 1712–1730. DOI: 10.1044/2024_JSLHR-23-00759. 38749007 PMC12379677

[ref3] Bachorowski J-A and Owren MJ (1995) Vocal expression of emotion: acoustic properties of speech are associated with emotional intensity and context. Psychological Science 6(4), 219–224. DOI: 10.1111/j.1467-9280.1995.tb00596.x.

[ref4] Baird A , Triantafyllopoulos A , Zänkert S , Ottl S , Christ L , Stappen L , Konzok J , Sturmbauer S , Meßner E-M , Kudielka BM , Rohleder N , Baumeister H and Schuller BW (2021) An evaluation of speech-based recognition of emotional and physiological markers of stress. Frontiers in Computer Science 3,750284. DOI: 10.3389/fcomp.2021.750284.

[ref5] Banse R and Scherer KR (1996) Acoustic profiles in vocal emotion expression. Journal of Personality and Social Psychology 70(3), 614–636. DOI: 10.1037/0022-3514.70.3.614. 8851745

[ref6] Benjamini Y and Hochberg Y (1995) Controlling the false discovery rate: a practical and powerful approach to multiple testing. Journal of the Royal Statistical Society. Series B (Methodological) 57(1), 289–300.

[ref7] Betti S , Lova RM , Rovini E , Acerbi G , Santarelli L , Cabiati M , Del Ry S and Cavallo F (2018) Evaluation of an integrated system of wearable physiological sensors for stress monitoring in working environments by using biological markers. IEEE Transactions on Bio-Medical Engineering 65(8), 1748–1758. DOI: 10.1109/TBME.2017.2764507. 29989933

[ref8] Bobade P and Vani M (2020) Stress detection with machine learning and deep learning using multimodal physiological data. In 2020 Second International Conference on Inventive Research in Computing Applications (ICIRCA), 51–57. DOI: 10.1109/ICIRCA48905.2020.9183244.

[ref9] Bodaghi D , Xue Q , Thomson S and Zheng X (2025) The effect of subglottic stenosis severity on vocal fold vibration and voice production in realistic laryngeal and airway geometries using fluid–Structure–Acoustics interaction simulation. Applied Sciences 15(3), 1168. DOI: 10.3390/app15031168. PMC12467110 41018146

[ref10] Bouhuys AL , Bloem GM and Groothuis TG (1995) Induction of depressed and elated mood by music influences the perception of facial emotional expressions in healthy subjects. Journal of Affective Disorders 33(4), 215–226. DOI: 10.1016/0165-0327(94)00092-n. 7790675

[ref11] Briefer EF (2012) Vocal expression of emotions in mammals: mechanisms of production and evidence. Journal of Zoology 288(1), 1–20. DOI: 10.1111/j.1469-7998.2012.00920.x.

[ref12] Brockmann M , Drinnan MJ , Storck C and Carding PN (2011) Reliable jitter and shimmer measurements in voice clinics: the relevance of vowel, gender, vocal intensity, and fundamental frequency effects in a typical clinical task. Journal of Voice: Official Journal of the Voice Foundation 25(1), 44–53. DOI: 10.1016/j.jvoice.2009.07.002. 20381308

[ref13] Buchanan TW , Laures-Gore JS and Duff MC (2014) Acute stress reduces speech fluency. Biological Psychology 97, 60–66. DOI: 10.1016/j.biopsycho.2014.02.005. 24555989

[ref14] Carrillo-González A , Atará-Piraquive Á.P , Camargo-Mendoza M , Hernández-Contreras JR and Cantor-Cutiva LC (2025) Colombian college professors work-related health: associations between stress and voice acoustics parameters. Revista de Investigación e Innovación en Ciencias de la Salud 7(1), 1–16. DOI: 10.46634/riics.333.

[ref15] Dahl KL and Stepp CE (2023) Effects of cognitive stress on voice acoustics in individuals with hyperfunctional voice disorders. American Journal of Speech–Language Pathology 32(1), 264–274. DOI: 10.1044/2022_AJSLP-22-00204. 36516470 PMC10023146

[ref16] Daviu N , Bruchas MR , Moghaddam B , Sandi C and Beyeler A (2019) Neurobiological links between stress and anxiety. Neurobiology of Stress 11, 100191. DOI: 10.1016/j.ynstr.2019.100191. 31467945 PMC6712367

[ref17] Dickerson SS and Kemeny ME (2004) Acute stressors and cortisol responses: a theoretical integration and synthesis of laboratory research. Psychological Bulletin 130(3), 355–391. DOI: 10.1037/0033-2909.130.3.355. 15122924

[ref18] Dietrich M and Verdolini Abbott K (2012) Vocal function in introverts and extraverts during a psychological stress reactivity protocol. Journal of Speech, Language, and Hearing Research : JSLHR 55(3), 973–987. DOI: 10.1044/1092-4388(2011/10-0344). 22232397

[ref19] Faul F , Erdfelder E , Lang A-G and Buchner A (2007) G*Power 3: a flexible statistical power analysis program for the social, behavioral, and biomedical sciences. Behavior Research Methods 39(2), 175–191. DOI: 10.3758/bf03193146. 17695343

[ref20] Fitch WT and Giedd J (1999) Morphology and development of the human vocal tract: a study using magnetic resonance imaging. The Journal of the Acoustical Society of America 106(3 Pt 1), 1511–1522. DOI: 10.1121/1.427148. 10489707

[ref69] Fukuda S and Morimoto K (2001). Lifestyle, stress and cortisol response: review II : lifestyle. Environmental health and preventive medicine 6(1), 15–21. DOI: 10.1007/BF02897304. 21432232 PMC2723649

[ref21] Gaikwad P and Venkatesan M (2024). Speech recognition-based prediction for mental health and depression: a review. In P. K. Jha , B. Tripathi , E. Natarajan , & H. Sharma (Hrsg.), (eds), Proceedings of Congress on Control, Robotics, and Mechatronics. Springer Nature, 13–24. DOI: 10.1007/978-981-99-5180-2_2.

[ref22] Giddens CL , Barron KW , Byrd-Craven J , Clark KF and Winter AS (2013) Vocal indices of stress: a review. Journal of Voice 27(3), 390.e21–390.e29. DOI: 10.1016/j.jvoice.2012.12.010. 23462686

[ref23] Grant JK , Forrest APM and Symington T (1957) The secretion of cortisol and corticosterone by the human adrenal cortex. Acta Endocrinologica 26(2), 195–203. DOI: 10.1530/acta.0.0260195. 13469084

[ref24] Hammen CL (2015) Stress and depression: old questions, new approaches. Current Opinion in Psychology 4, 80–85. DOI: 10.1016/j.copsyc.2014.12.024.

[ref25] Hansen JHL and Patil S (2007a) Speech under stress: analysis, modeling and recognition. In Müller C (Hrsg.) (ed), Speaker Classification I: Fundamentals, Features, and Methods, Springer, pp. 108–137. DOI: 10.1007/978-3-540-74200-5_6.

[ref26] Hellhammer DH , Wüst S and Kudielka BM (2009) Salivary cortisol as a biomarker in stress research. Psychoneuroendocrinology 34(2), 163–171. DOI: 10.1016/j.psyneuen.2008.10.026. 19095358

[ref27] Holmqvist S , Santtila P , Lindström E , Sala E and Simberg S (2013) The association between possible stress markers and vocal symptoms. Journal of Voice: Official Journal of the Voice Foundation 27(6), 787.e1–787.e10. DOI: 10.1016/j.jvoice.2013.06.012. 23880012

[ref28] Huttunen K , Keränen H , Väyrynen E , Pääkkönen R and Leino T (2011) Effect of cognitive load on speech prosody in aviation: evidence from military simulator flights. Applied Ergonomics 42(2), 348–357. DOI: 10.1016/j.apergo.2010.08.005. 20832770

[ref29] Izawa S , Nakamura-Taira N and Yamada KC (2016) Stress underestimation and mental health outcomes in male Japanese workers: a 1-year prospective study. International Journal of Behavioral Medicine 23(6), 664–669. DOI: 10.1007/s12529-016-9557-8. 26979425

[ref30] Kappen M , van der Donckt J , Vanhollebeke G , Allaert J , Degraeve V , Madhu N , Van Hoecke S and Vanderhasselt M-A (2022) Acoustic speech features in social comparison: how stress impacts the way you sound. Scientific Reports 12(1), 22022. DOI: 10.1038/s41598-022-26375-9. 36539505 PMC9767914

[ref31] König A , Linz N , Zeghari R , Klinge X , Tröger J , Alexandersson J and Robert P (2019) Detecting apathy in older adults with cognitive disorders using automatic speech analysis. Journal of Alzheimer’s Disease 69(4), 1183–1193. DOI: 10.3233/JAD-181033. 31127764

[ref32] König A , Riviere K , Linz N , Elbaum J , Fabre R and Robert P (2021) Measuring stress in health professionals over the phone using automatic speech analysis during COVID-19 pandemic: observational study. Journal of Medical Internet Research 23(4), e24191. DOI: 10.2196/24191. 33739930 PMC8057197

[ref33] Ladefoged P (1996) Elements of acoustic phonetics, 2nd edn. University of Chicago Press.

[ref34] Ladefoged P and Johnson K (2011) A course in phonetics, 6th edn. Michael Rosenberg.

[ref35] Laukkanen A-M , Ilomäki I , Leppänen K and Vilkman E (2008) Acoustic measures and self-reports of vocal fatigue by female teachers. Journal of Voice: Official Journal of the Voice Foundation 22(3), 283–289. DOI: 10.1016/j.jvoice.2006.10.001. 17134877

[ref68] Lee DY , Kim E and Choi MH (2015) Technical and clinical aspects of cortisol as a biochemical marker of chronic stress. BMB reports 48(4), 209–216. DOI: 10.5483/bmbrep.2015.48.4.275. 25560699 PMC4436856

[ref36] Lehrl S (2005) Mehrfachwahl-wortschatz-intelligenztest: MWT-B (5. Aufl.). Balingen: Spitta.

[ref37] Liu JJW , Ein N , Gervasio J , Vickers K and Zhou X-J (2019) The efficacy of stress reappraisal interventions on stress responsivity: a meta-analysis and systematic review of existing evidence. PLoS ONE 14(2), e0212854. DOI: 10.1371/journal.pone.0212854. 30811484 PMC6392321

[ref38] Marieb EN and Hoehn K (2007) Human anatomy & physiology/ Elaine N. Marieb, Katja Hoehn. Seventh edn. Pearson Benjamin Cummings.

[ref39] Mattei A , Legou T , Cardeau A , Le Goff J , Lagier A and Giovanni A (2019) Acoustic correlates of vocal effort: external factors and personality traits. European Annals of Otorhinolaryngology, Head and Neck Diseases 136(3), 151–154. DOI: 10.1016/j.anorl.2019.02.010. 30880033

[ref40] Nakagawa S , Asakawa K and Wang L (2007) Speaker recognition by combining MFCC and phase information. Proceedings of the Annual Conference of the International Speech Communication Association, INTERSPEECH. 2. 2005-2008. DOI: 10.21437/Interspeech.2007-161.

[ref41] Nath RK and Thapliyal H (2021) Smart wristband-based stress detection framework for older adults with cortisol as stress biomarker. IEEE Transactions on Consumer Electronics 67(1), 30–39. DOI: 10.1109/TCE.2021.3057806.

[ref42] O’Connor DB , Thayer JF and Vedhara K (2021) Stress and health: a review of psychobiological processes. Annual Review of Psychology 72(1), 663–688. DOI: 10.1146/annurev-psych-062520-122331. 32886587

[ref71] Paulmann S , Furnes D , Bøkenes AM and Cozzolino, PJ (2016) How psychological stress affects emotional prosody. PloS one 11(11), e0165022. DOI: 10.1371/journal.pone.0165022. 27802287 PMC5089770

[ref43] Pêgo JM , Sousa JC , Almeida O and Sousa N (2010) Stress and the neuroendocrinology of anxiety disorders. In Stein MB and Steckler T (Hrsg.) (eds), Behavioral neurobiology of anxiety and its treatment. Springer, pp. 97–118. DOI: 10.1007/7854_2009_13. 21309108

[ref44] Pisanski K and Sorokowski P (2021) Human stress detection: cortisol levels in stressed speakers predict voice-based judgments of stress. Perception 50(1), 80–87. DOI: 10.1177/0301006620978378. 33302780

[ref70] Pritchard BT , Stanton W , Lord R , Petocz P , Pepping G-J (2017) Factors affecting measurement of salivary cortisol and secretory immunoglobulin A in field studies of athletes. Frontiers in endocrinology 8, 168. DOI: 10.3389/fendo.2017.00168. 28790976 PMC5522838

[ref45] Protopapas A and Lieberman P (1997) Fundamental frequency of phonation and perceived emotional stress. The Journal of the Acoustical Society of America 101(4), 2267–2277. DOI: 10.1121/1.418247. 9104028

[ref46] Rajasekaran P , Doddington G and Picone J (1986) Recognition of speech under stress and in noise. In ICASSP ’86. IEEE International Conference on Acoustics, Speech, and Signal Processing, Vol. 11, pp. 733–736. DOI: 10.1109/ICASSP.1986.1169207.

[ref47] Reitan RM (1955) The relation of the trail making test to organic brain damage. Journal of Consulting Psychology 19(5), 393–394. DOI: 10.1037/h0044509. 13263471

[ref48] Robin J , Harrison JE , Kaufman LD , Rudzicz F , Simpson W and Yancheva M (2020) Evaluation of speech-based digital biomarkers: review and recommendations. Digital Biomarkers 4(3), 99–108. DOI: 10.1159/000510820. 33251474 PMC7670321

[ref49] Schamborg S , Tully RJ and Browne KD (2016) The use of the state–Trait anger expression inventory–II with forensic populations: a psychometric critique. International Journal of Offender Therapy and Comparative Criminology 60(11), 1239–1256. DOI: 10.1177/0306624X15577932. 25899599

[ref50] Scherer KR (1986) Vocal affect expression: a review and a model for future research. Psychological Bulletin 99(2), 143–165. DOI: 10.1037/0033-2909.99.2.143. 3515381

[ref51] Schewski L , Doss MM , Beldi G , Keller S and Biassoni F (2025) Measuring negative emotions and stress through acoustic correlates in speech: a systematic review. PLOS ONE 20(7), e0328833. DOI: 10.1371/journal.pone.0328833. 40705747 PMC12289014

[ref52] Schwabe L , Haddad L and Schachinger H (2008) HPA axis activation by a socially evaluated cold-pressor test. Psychoneuroendocrinology 33(6), 890–895. DOI: 10.1016/j.psyneuen.2008.03.001. 18403130

[ref53] Schwabe L and Schächinger H (2018) Ten years of research with the socially evaluated cold pressor test: data from the past and guidelines for the future. Psychoneuroendocrinology 92, 155–161. DOI: 10.1016/j.psyneuen.2018.03.010. 29573884

[ref54] Slavich GM (2019) Stressnology: the primitive (and problematic) study of life stress exposure and pressing need for better measurement. Brain, Behavior, and Immunity 75, 3–5. DOI: 10.1016/j.bbi.2018.08.011. 30236597 PMC6279572

[ref55] Sondhi S , Khan M , Vijay R and K. Salhan A (2015) Vocal indicators of emotional stress. International Journal of Computer Applications 122(15), 38–43. DOI: 10.5120/21780-5056.

[ref56] Spielberger CD , Sydeman SJ , Owen AE and Marsh BJ (1999) Measuring anxiety and anger with the state–trait anxiety inventory (STAI) and the state–trait anger expression inventory (STAXI). In The use of psychological testing for treatment planning and outcomes assessment, 2nd edn. Lawrence Erlbaum Associates Publishers, pp. 993–1021.

[ref57] Stalder T , Kirschbaum C , Kudielka BM , Adam EK , Pruessner JC , Wüst S , Dockray S , Smyth N , Evans P , Hellhammer DH , Miller R , Wetherell MA , Lupien SJ and Clow A (2016) Assessment of the cortisol awakening response: expert consensus guidelines. Psychoneuroendocrinology 63, 414–432. DOI: 10.1016/j.psyneuen.2015.10.010. 26563991

[ref58] Tafet GE and Nemeroff CB (2016) The links between stress and depression: psychoneuroendocrinological, genetic, and environmental interactions. The Journal of Neuropsychiatry and Clinical Neurosciences 28(2), 77–88. DOI: 10.1176/appi.neuropsych.15030053. 26548654

[ref59] Teixeira JP , Oliveira C and Lopes C (2013) Vocal acoustic analysis – jitter, shimmer and HNR parameters. Procedia Technology 9, 1112–1122. DOI: 10.1016/j.protcy.2013.12.124.

[ref60] Tolkmitt FJ and Scherer KR (1986) Effect of experimentally induced stress on vocal parameters. Journal of Experimental Psychology. Human Perception and Performance 12(3), 302–313. DOI: 10.1037//0096-1523.12.3.302. 2943858

[ref61] van der Woerd B , Wu M , Parsa V , Doyle PC and Fung K (2020) Evaluation of acoustic analyses of voice in nonoptimized conditions. Journal of Speech, Language, and Hearing Research: JSLHR 63(12), 3991–3999. DOI: 10.1044/2020_JSLHR-20-00212. 33186510

[ref62] Van Lierde K , Van Heule S , De Ley S , Mertens E and Claeys S (2009) Effect of psychological stress on female vocal quality. A multiparameter approach. Folia Phoniatrica et Logopaedica: Official Organ of the International Association of Logopedics and Phoniatrics (IALP) 61(2), 105–111. DOI: 10.1159/000209273. 19299899

[ref63] Vinutha HP , Poornima B and Sagar BM (2018) Detection of outliers using interquartile range technique from intrusion dataset. In Satapathy SC , Tavares JMRS , Bhateja V and Mohanty JR (Hrsg.), (eds), Information and decision sciences, Springer, pp. 511–518, DOI: 10.1007/978-981-10-7563-6_53.

[ref64] Wilcoxon F (1945) Individual comparisons by ranking methods. Biometrics Bulletin 1(6), 80–83. 18903631

[ref65] Zellner B (1994) Pauses and the temporal structure of speech. In Fundamentals of speech synthesis and speech recognition. John Wiley, pp. 41–62.

[ref66] Zhang Z (2021) Contribution of laryngeal size to differences between male and female voice production. The Journal of the Acoustical Society of America 150(6), 4511–4521. DOI: 10.1121/10.0009033. 34972311 PMC8716178

[ref67] Zhang Z (2024) Interaction effects in laryngeal and respiratory control of the voice source and vocal fold contact pressure. The Journal of the Acoustical Society of America 156(6), 4326–4335. DOI: 10.1121/10.0034708. 39740048 PMC11693206

