# Supplementary material for: Voice as objective biomarker of stress: association of speech features and cortisol
Source: Acta Neuropsychiatr. 2025 Sep 3;37:e84. doi: 10.1017/neu.2025.10037 (PMC13130297; doi:10.1017/neu.2025.10037)
Supplement: Menne et al. supplementary material [file S0924270825100379sup001.docx]

**Supplementary Materials**

**Full sentence list:**

| **German Original** | **English Translation** |
| --- | --- |
| Der Lappen liegt auf dem Eisschrank. | The rag is lying on the freezer. |
| Das will sie am Mittwoch abgeben. | She wants to hand that in on Wednesday. |
| Heute Abend könnte ich es ihm sagen. | I could tell him this evening. |
| Das schwarze Stück Papier befindet sich da oben neben dem Holzstück. | The black piece of paper is up there next to the piece of wood. |
| In sieben Stunden wird es soweit sein. | In seven hours it will be time. |
| Was sind denn das für Tüten, die da unter dem Tisch stehen? | What kind of bags are those standing under the table? |
| Sie haben es gerade hochgetragen und jetzt gehen sie wieder runter. | They just carried it upstairs and now they're going down again. |
| An den Wochenenden bin ich jetzt immer nach Hause gefahren und habe Agnes besucht. | On weekends, I’ve been going home and visiting Agnes. |
| Ich will das eben wegbringen und dann mit Karl was trinken gehen. | I just want to drop this off and then go have a drink with Karl. |
| Die wird auf dem Platz sein, wo wir sie immer hinlegen. | It will be in the spot where we always put it. |
| Ich weiß genau, was ich auf dem Schiff gesagt habe. | I know exactly what I said on the ship. |
| Sie hat gesagt, sie überlässt ihn mir, sie würde mir sogar helfen. | She said she’s leaving him to me, she’d even help me. |
| Beeil dich, wir verpassen sonst noch den Bus! | Hurry up or we’ll miss the bus! |
| Das Frühstück war sehr lecker, aber die Bedienung war wirklich unfreundlich. | The breakfast was delicious, but the service was really unfriendly. |
| Am Donnerstag hat es stundenlang in Strömen geregnet. | It rained in torrents for hours on Thursday. |
| Kannst du mir bitte helfen, den Einkauf hoch zu tragen? | Can you please help me carry the groceries upstairs? |
| Dieter ist nicht mehr er selbst, seit er vierzig geworden ist. | Dieter hasn’t been himself since he turned forty. |
| Ich muss jetzt erst mal auf die Post, danach können wir weiter reden. | I have to go to the post office first, then we can continue talking. |
| Er scheint zu hoffen, dass er dann besser da steht. | He seems to hope that he'll be in a better position then. |
| Franz und Marie sind heute nicht da, sie haben diese Woche Urlaub. | Franz and Marie aren’t here today, they’re on vacation this week. |

**Supp. Table 1:** Categories of analyzed categories with associated speech features

| Feature group | Feature Unit | Feature Name | Feature Explanation |
| --- | --- | --- | --- |
| Energy | *Amplitude perturbation quotient (%)* | apq3_shimmer | Three-point period amplitude perturbation quotient, a measure of short-term changes in voice loudness calculated from three consecutive vocal cycles. |
|  |  | apq5_shimmer | Five-point period amplitude perturbation quotient. |
|  |  | apq11_shimmer | Eleven-point period amplitude perturbation quotient. |
|  | *Average absolute difference in amplitude between cycles (%)* | dda_shimmer | Dynamic Decline Amplitude Shimmer (average absolute difference in loudness between consecutive vocal cycles). |
|  | *Harmonic-to-Noise Ratio (dB)* | hnr_mean | Average Harmonic-to-Noise Ratio (HNR) in decibels, estimated using cepstral analysis to measure the balance between harmonic (voiced) and noise components in the voice. |
|  |  | hnr_sd | Standard deviation of hnr_mean. |
|  | *Cycle-to-cycle amplitude variation (%)* | local_shimmer | Cycle-to-cycle variation in voice loudness, expressed as a percentage of the average amplitude. |
|  | *Loudness (dB)* | loudness_mean | Mean speech loudness. |
|  |  | loudness_sd | Standard deviation of loudness_mean. |
|  | *Number of peaks per second (Hz)* | rate_loudness_peaks | Frequency of loudness peaks in a given time period. |
|  | *Shimmer in dB (dB)* | shimmer_local_dB_mean | Average cycle-to-cycle variation in voice loudness, measured in decibels. |
|  |  | shimmer_local_dB_sd | Standard deviation of difference of shimmer_local_dB_mean. |
| Frequency | *Cycle-to-cycle pitch difference (Hz)* | ddp_jitter | Dynamic Decline Perturbation in jitter (average absolute difference in pitch (F0) between consecutive vocal cycles). |
|  | *Range of pitch (Hz)* | f0_range | Range of vocal fold vibration frequency (F0). |
|  | *Formant bandwidth (Hz)* | f1_bandwidth_mean | Mean bandwidth of F1 formant. |
|  |  | f1_bandwidth_sd | Standard deviation of f1_bandwidth_mean. |
|  | *Formant frequency (Hz)* | f1_frequency_mean | Mean frequency of F1 formant. |
|  |  | f1_frequency_sd | Standard deviation of f1_frequency_mean. |
|  | *Formant bandwidth (Hz)* | f2_bandwidth_mean | Mean bandwidth of F2 formant. |
|  |  | f2_bandwidth_sd | Standard deviation of f2_bandwidth_mean. |
|  | *Formant frequency (Hz)* | f2_frequency_mean | Mean frequency of F2 formant. |
|  |  | f2_frequency_sd | Standard deviation of f2_frequency_mean. |
|  | *Formant bandwidth (Hz)* | f3_bandwidth_mean | Mean bandwidth of F3 formant. |
|  |  | f3_bandwidth_sd | Standard deviation of f3_bandwidth_mean. |
|  | *Formant frequency (Hz)* | f3_frequency_mean | Mean frequency of F3 formant. |
|  |  | f3_frequency_sd | Standard deviation of f3_frequency_mean. |
|  | *Cycle-to-cycle pitch variation (Hz)* | jitter_local_mean | Average cycle-to-cycle variation in pitch (F0), perceived as voice irregularity. |
|  |  | jitter_local_sd | Standard deviation of jitter_local_mean. |
|  |  | local_absolute_jitter | Cycle-to-cycle variation in pitch (F0). |
|  | *Pitch (Hz)* | pitch_max | Maximum pitch. |
|  |  | pitch_mean | Mean pitch. |
|  |  | pitch_min | Minimum pitch. |
|  |  | pitch_range | Range of pitch. |
|  |  | pitch_std | Standard deviation of pitch. |
|  | *Pitch perturbation quotient (%)* | ppq5_jitter | Five-point period perturbation quotient (pitch (F0) variation measured across five consecutive vocal cycles). |
|  |  | rap_jitter | Relative Average Perturbation jitter (average relative variation in pitch (F0) between consecutive vocal cycles.) |
|  | *Frequency fluctuation range (Hz)* | vocal_tremor | Intensity of low-frequency pitch (F0) fluctuations (1.5-15 Hz). |
| Spectral | *Energy ratio (unitless)* | alpha_ratio_mean | Average ratio of energy between low (50-1000 Hz) and high (1-5 kHz) frequencies. |
|  |  | alpha_ratio_sd | Standard deviation of alpha_ratio_mean. |
|  | *MFCC coefficients (unitless)* | average_mfccs_1 | Average of the Mel-Frequency-Cepstral-Coefficient 1 (Measurement to capture fundamental frequency characteristics of human speech). |
|  |  | average_mfccs_2 | Average of the Mel-Frequency-Cepstral-Coefficient 2. |
|  |  | average_mfccs_3 | Average of the Mel-Frequency-Cepstral-Coefficient 3. |
|  |  | average_mfccs_4 | Average of the Mel-Frequency-Cepstral-Coefficient 4. |
|  | *Relative energy (unitless)* | f1_relative_energy_mean | Average relative energy of the first formant (F1), reflecting the prominence of F1 resonance. |
|  |  | f1_relative_energy_sd | Standard deviation of f1_relative_energy_mean. |
|  |  | f2_relative_energy_mean | Average relative energy of the second formant (F2), reflecting the prominence of F2 resonance. |
|  |  | f2_relative_energy_sd | Standard deviation of f2_relative_energy_mean. |
|  |  | f3_relative_energy_mean | Average relative energy of the third formant (F3), reflecting the prominence of F3 resonance. |
|  |  | f3_relative_energy_sd | Standard deviation of f3_relative_energy_mean. |
|  | *Energy ratio (dB)* | h1_a3_harmonic_difference_mean | Average energy ratio between the first F0 harmonic (H1) and the strongest harmonic in the third formant range (A3). |
|  |  | h1_a3_harmonic_difference_sd | Standard deviation of h1_a3_harmonic_difference_mean. |
|  |  | h1_h2_harmonic_difference_mean | Average energy ratio between the first F0 harmonic (H1) and the strongest harmonic in the second formant range (H2). |
|  |  | h1_h2_harmonic_difference_sd | Standard deviation of h1_h2_harmonic_difference_mean. |
|  | *Spectral energy ratio (unitless)* | hammarberg_index_mean | Ratio of energy between higher (2–5 kHz) and lower (0.5–2 kHz) frequency bands. Parameter to assess the spectral balance of a voice. |
|  |  | hammarberg_index_sd | Standard deviation of Hammarberg Index. |
|  | *Spectral slope (dB/Hz)* | spectral_slope_0_500_mean | Average slope of the logarithmic power spectrum in the 0-500 Hz range. |
|  |  | spectral_slope_0_500_sd | Standard deviation of spectral_slope_0_500_mean. |
|  |  | spectral_slope_500_1500_mean | Average slope of the logarithmic power spectrum in the 500-1500 Hz range. |
|  |  | spectral_slope_500_1500_sd | Standard deviation of Slope V500-1500. |
| Temporal | *Total recording length (seconds)* | duration | Length of audio recording. |
|  | *Count (unitless)* | number_of_pauses | Number of pauses in between speech segments based on speech intervals. |
|  | *Pause duration (seconds)* | pause_durations_mean | Mean length of pauses. |
|  |  | pause_durations_sd | Standard deviation of pause duration. |
|  |  | pause_durations_sum | Sum of pause lengths over whole utterance. |
|  | *Ratio (unitless)* | pause_rate | Frequency of pauses within a spoken utterance. |
|  |  | speech_ratio | Ratio of utterance to duration. |
|  | *Utterance duration (seconds)* | utterance_durations_mean | Mean length of utterances. |
|  |  | utterance_durations_sd | Standard deviation of utterance durations. |
|  |  | utterance_durations_sum | Sum of utterance durations. |

**Supp. Table 2**: Difference in Speech features before and after intervention. Features that remained significant after correction are highlighted in bold. Before and after indicate if the feature value increased or decreased in value after exposure to cold/warm water. Features are organized by feature type and then alphabetically.

|  |  | **WARM** | | | **COLD** | | |
| --- | --- | --- | --- | --- | --- | --- | --- |
| **Feature Type** | **Feature** | **Effect Size** | **Before v. After** | **p(adj)** | **Effect Size** | **Before v. After** | **p(adj)** |
| Energy | apq3_shimmer | -0.053 | < | 0.228 | -0.006 | < | 0.957 |
|  | apq5_shimmer | -0.033 | < | 0.465 | -0.027 | < | 0.59 |
|  | dda_shimmer | -0.053 | < | 0.228 | -0.006 | < | 0.957 |
|  | **hnr_mean** | **-0.229** | **<** | **0.00** | -0.067 | < | 0.102 |
|  | **hnr_sd** | **-0.244** | **>** | **0.00** | -0.07 | > | 0.091 |
|  | local_dB_shimmer | -0.024 | < | 0.614 | -0.039 | < | 0.377 |
|  | local_shimmer | -0.046 | < | 0.298 | -0.031 | < | 0.508 |
|  | **loudness_mean** | -0.077 | > | 0.071 | **-0.128** | **<** | **0.001** |
|  | **loudness_sd** | **-0.131** | **>** | **0.001** | -0.057 | < | 0.17 |
|  | **rate_loudness_peaks** | **-0.112** | **<** | **0.009** | **-0.159** | **<** | **<0.001** |
| Frequency | ddp_jitter | -0.031 | < | 0.469 | -0.075 | < | 0.083 |
|  | **f1_bandwidth_mean** | **-0.167** | **<** | **0.00** | -0.009 | > | 0.957 |
|  | **f1_bandwidth_sd** | **-0.118** | **<** | **0.003** | -0.06 | > | 0.158 |
|  | f1_frequency_mean | -0.053 | < | 0.228 | -0.005 | > | 0.969 |
|  | f1_frequency_sd | -0.078 | < | 0.07 | -0.071 | > | 0.091 |
|  | f2_bandwidth_mean | -0.054 | < | 0.228 | -0.001 | < | 0.986 |
|  | f2_bandwidth_sd | -0.071 | < | 0.104 | -0.081 | > | 0.058 |
|  | **f2_frequency_mean** | **-0.225** | **<** | **0.00** | -0.07 | > | 0.091 |
|  | **f2_frequency_sd** | -0.041 | < | 0.36 | **-0.085** | **>** | **0.047** |
|  | **f3_bandwidth_mean** | -0.048 | > | 0.275 | **-0.218** | **<** | **<0.001** |
|  | f3_bandwidth_sd | -0.015 | > | 0.746 | -0.056 | < | 0.17 |
|  | **f3_frequency_mean** | **-0.169** | **<** | **0.00** | -0.081 | < | 0.059 |
|  | f3_frequency_sd | -0.065 | < | 0.139 | -0.001 | > | 0.986 |
|  | local_absolute_jitter | -0.037 | < | 0.421 | -0.049 | < | 0.237 |
|  | local_jitter | -0.041 | < | 0.357 | -0.073 | < | 0.083 |
|  | pitch_max | -0.035 | > | 0.434 | -0.034 | > | 0.464 |
|  | **pitch_mean** | **-0.115** | **>** | **0.004** | -0.033 | < | 0.477 |
|  | **pitch_min** | **-0.083** | **>** | **0.05** | -0.017 | > | 0.796 |
|  | pitch_range | -0.036 | < | 0.427 | -0.012 | > | 0.928 |
|  | **pitch_std** | **-0.16** | **<** | **0.00** | -0.069 | < | 0.094 |
|  | ppq5_jitter | -0.021 | < | 0.66 | -0.074 | < | 0.083 |
|  | rap_jitter | -0.031 | < | 0.469 | -0.075 | < | 0.083 |
|  | vocal_tremor | -0.002 | > | 0.967 | -0.01 | > | 0.954 |
| Spectral | alpha_ratio_mean | -0.044 | > | 0.32 | -0.009 | < | 0.957 |
|  | alpha_ratio_sd | -0.06 | > | 0.178 | -0.056 | > | 0.17 |
|  | **average_mfccs_1** | **-0.254** | **>** | **0.00** | -0.001 | < | 0.986 |
|  | **average_mfccs_2** | **-0.136** | **<** | **0.001** | **-0.088** | **<** | **0.038** |
|  | **average_mfccs_3** | **-0.096** | **<** | **0.018** | **-0.229** | **>** | **<0.001** |
|  | **average_mfccs_4** | -0.063 | > | 0.159 | **-0.131** | **>** | **0.001** |
|  | **f1_relative_energy_mean** | **-0.129** | **>** | **0.001** | -0.004 | > | 0.986 |
|  | f1_relative_energy_sd | -0.02 | > | 0.661 | -0.047 | > | 0.259 |
|  | f2_relative_energy_mean | -0.032 | > | 0.469 | -0.029 | > | 0.541 |
|  | f2_relative_energy_sd | -0.003 | < | 0.967 | -0.058 | > | 0.167 |
|  | **f3_relative_energy_mean** | -0.058 | < | 0.193 | **-0.17** | **>** | **<0.001** |
|  | f3_relative_energy_sd | -0.006 | > | 0.906 | -0.018 | < | 0.794 |
|  | **h1_a3_harmonic_difference_mean** | **-0.13** | **<** | **0.001** | **-0.162** | **>** | **<0.001** |
|  | **h1_a3_harmonic_difference_sd** | -0.001 | > | 0.967 | **-0.117** | **>** | **0.003** |
|  | **h1_h2_harmonic_difference_mean** | -0.02 | > | 0.661 | **-0.09** | **>** | **0.035** |
|  | **h1_h2_harmonic_difference_sd** | **-0.135** | **>** | **0.001** | **-0.145** | **>** | **<0.001** |
|  | **hammarberg_index_mean** | **-0.187** | **>** | **0.00** | -0.073 | < | 0.083 |
|  | hammarberg_index_sd | -0.052 | < | 0.233 | 0.008 | > | 0.957 |
|  | **spectral_slope_0_500_mean** | **-0.122** | **>** | **0.003** | -0.007 | > | 0.957 |
|  | **spectral_slope_0_500_sd** | -0.059 | > | 0.19 | **-0.125** | **>** | **0.001** |
|  | **spectral_slope_500_1500_mean** | **-0.102** | **>** | **0.012** | -0.054 | > | 0.18 |
|  | spectral_slope_500_1500_sd | -0.017 | > | 0.712 | -0.055 | > | 0.18 |
| Temporal | **duration** | **-0.414** | **>** | **0.00** | **-0.383** | **>** | **<0.001** |
|  | **number_of_pauses** | -0.33 | > | 0.106 | **-0.428** | **>** | **<0.001** |
|  | **pause_durations_mean** | -0.12 | > | 0.003 | -0.063 | > | 0.165 |
|  | **pause_durations_sd** | **-0.106** | **>** | **0.009** | **-0.119** | **>** | **0.002** |
|  | **pause_durations_sum** | **-0.189** | **>** | **0.00** | **-0.193** | **>** | **<0.001** |
|  | pause_rate | -0.008 | > | 0.888 | -0.003 | > | 0.986 |
|  | **speech_ratio** | **-0.097** | **<** | **0.017** | -0.002 | < | 0.986 |
|  | **utterance_durations_mean** | **-0.125** | **>** | **0.003** | -0.078 | < | 0.083 |
|  | utterance_durations_sd | -0.011 | > | 0.82 | -0.058 | < | 0.165 |
|  | **utterance_durations_sum** | **-0.241** | **>** | **0.00** | **-0.26** | **>** | **<0.001** |

**Supp. Table 3:** Associations of acoustic features with cortisol levels across conditions. The table presents correlation coefficients (corr), effect sizes (ES), and adjusted p-values (p-adj) for the overall population. Sex-specific correlations are indicated separately for males (M) and females (F), with positive (+) or negative (-) directions. Fisher’s Z-test results, including Z-values and corresponding p-values, are reported under the "Sex Comparison" header to assess differences between sexes. Results are shown for both cold and warm conditions.

| **Cortisol Levels Difference Exposure to Stress Condition** | | **COLD** | | | | | | | **WARM** | | | | | | |
| --- | --- | --- | --- | --- | --- | --- | --- | --- | --- | --- | --- | --- | --- | --- | --- |
|  |  | ***Overall*** | | | ***Sex Comparison*** | | | | ***Overall*** | | | ***Sex Comparison*** | | | |
| **Feature Type** | **Feature** | **Corr** | **ES** | **p-adj** | **M** | **F** | **Z** | **p** | **Corr** | **ES** | **p-adj** | **M** | **F** | **Z** | **p** |
| Energy | apq3_shimmer | -0.02 | 0.03 | 0.79 | - | + | -2.32 | 0.02 | -0.03 | 0.07 | 0.73 | - | + | -2.32 | 0.02 |
|  | apq5_shimmer | 0.04 | 0.08 | 0.51 | - | + | -2.03 | 0.04 | 0.01 | 0.02 | 0.91 | - | + | -2.03 | 0.04 |
|  | dda_shimmer | -0.02 | 0.03 | 0.79 | - | + | -2.32 | 0.02 | -0.03 | 0.07 | 0.73 | - | + | -2.32 | 0.02 |
|  | hnr_mean | 0.05 | 0.1 | 0.37 | + | + | -1.4 | 0.16 | 0.04 | 0.08 | 0.72 | + | - | -1.4 | 0.16 |
|  | hnr_sd | -0.06 | 0.12 | 0.22 | - | - | 1.7 | 0.09 | 0 | 0.01 | 0.94 | - | + | 1.7 | 0.09 |
|  | local_shimmer | 0.01 | 0.01 | 0.92 | - | + | -3.37 | 0 | -0.02 | 0.03 | 0.9 | - | + | -3.37 | <0.001 |
|  | **loudness_mean** | **-0.18** | **0.36** | **0** | **-** | **-** | **-0.18** | **0.86** | **-0.01** | **0.02** | **0.91** | **-** | **+** | **-0.18** | **0.86** |
|  | loudness_sd | 0 | 0.01 | 0.96 | + | - | 0.31 | 0.76 | 0.11 | 0.21 | 0.06 | + | + | 0.31 | 0.76 |
|  | rate_loudness_peaks | -0.03 | 0.05 | 0.69 | - | + | -1.67 | 0.1 | 0 | 0 | 0.98 | - | + | -1.67 | 0.1 |
|  | local_dB_shimmer | -0.01 | 0.02 | 0.83 | - | + | -3.26 | 0 | -0.01 | 0.02 | 0.91 | - | - | -3.26 | <0.001 |
| Frequency | **ddp_jitter** | **-0.1** | **0.2** | **0.03** | **-** | **-** | **-0.19** | **0.85** | **0.06** | **0.13** | **0.3** | **+** | **+** | **-0.19** | **0.85** |
|  | f1_bandwidth_mean | -0.02 | 0.04 | 0.79 | - | + | -2.43 | 0.02 | -0.04 | 0.09 | 0.64 | - | - | -2.43 | 0.02 |
|  | f1_bandwidth_sd | -0.03 | 0.05 | 0.69 | - | + | -1.72 | 0.08 | -0.03 | 0.06 | 0.77 | - | - | -1.72 | 0.08 |
|  | f1_frequency_mean | 0.01 | 0.01 | 0.92 | + | + | -0.09 | 0.93 | -0.01 | 0.03 | 0.9 | - | - | -0.09 | 0.93 |
|  | f1_frequency_sd | 0.04 | 0.08 | 0.51 | + | + | -1.37 | 0.17 | -0.02 | 0.05 | 0.79 | - | - | -1.37 | 0.17 |
|  | f2_bandwidth_mean | -0.05 | 0.09 | 0.41 | + | - | 1.55 | 0.12 | -0.02 | 0.05 | 0.79 | - | + | 1.55 | 0.12 |
|  | f2_bandwidth_sd | -0.02 | 0.03 | 0.8 | + | - | 1.66 | 0.1 | 0.03 | 0.06 | 0.76 | - | + | 1.66 | 0.1 |
|  | f2_frequency_mean | 0.01 | 0.03 | 0.8 | - | + | -1.91 | 0.06 | -0.07 | 0.13 | 0.3 | - | - | -1.91 | 0.06 |
|  | f2_frequency_sd | 0.08 | 0.15 | 0.1 | + | + | -0.87 | 0.39 | -0.03 | 0.06 | 0.77 | + | - | -0.87 | 0.39 |
|  | f3_bandwidth_mean | -0.06 | 0.12 | 0.22 | + | - | 3.84 | 0 | 0.12 | 0.23 | 0.03 | - | + | 3.84 | <0.001 |
|  | f3_bandwidth_sd | -0.05 | 0.11 | 0.31 | + | - | 1.99 | 0.05 | 0.06 | 0.11 | 0.4 | + | + | 1.99 | 0.05 |
|  | f3_frequency_mean | 0.04 | 0.07 | 0.56 | + | + | -0.91 | 0.37 | -0.06 | 0.13 | 0.3 | + | - | -0.91 | 0.37 |
|  | f3_frequency_sd | -0.05 | 0.1 | 0.35 | + | - | 1.83 | 0.07 | 0.05 | 0.09 | 0.55 | + | + | 1.83 | 0.07 |
|  | **local_absolute_jitter** | **-0.1** | **0.21** | **0.02** | **-** | **-** | **-0.97** | **0.33** | **0.08** | **0.15** | **0.3** | **+** | **-** | **-0.97** | **0.33** |
|  | pitch_max | -0.01 | 0.02 | 0.83 | + | - | 1.27 | 0.2 | 0.03 | 0.06 | 0.76 | + | + | 1.27 | 0.2 |
|  | pitch_mean | -0.06 | 0.12 | 0.22 | + | - | 2.65 | 0.01 | 0.01 | 0.01 | 0.91 | - | + | 2.65 | 0.01 |
|  | pitch_min | -0.02 | 0.05 | 0.7 | + | - | 1.88 | 0.06 | -0.01 | 0.01 | 0.91 | + | - | 1.88 | 0.06 |
|  | pitch_range | 0.01 | 0.03 | 0.8 | - | + | -0.7 | 0.48 | 0.05 | 0.1 | 0.52 | + | + | -0.7 | 0.48 |
|  | pitch_std | 0.03 | 0.05 | 0.69 | + | + | 0.13 | 0.9 | -0.02 | 0.04 | 0.83 | - | + | 0.13 | 0.9 |
|  | **ppq5_jitter** | **-0.1** | **0.2** | **0.02** | **-** | **-** | **-0.46** | **0.65** | **0.1** | **0.2** | **0.08** | **+** | **+** | **-0.46** | **0.65** |
|  | **rap_jitter** | **-0.1** | **0.2** | **0.03** | **-** | **-** | **-0.19** | **0.85** | **0.06** | **0.13** | **0.3** | **+** | **+** | **-0.19** | **0.85** |
|  | **vocal_tremor** | **0.09** | **0.18** | **0.05** | **+** | **+** | **2.48** | **0.01** | **-0.02** | **0.05** | **0.79** | **-** | **-** | **2.48** | **0.01** |
|  | local_jitter | -0.1 | 0.19 | 0.03 | - | - | -0.5 | 0.62 | 0.07 | 0.13 | 0.3 | + | + | -0.5 | 0.62 |
| Spectral | **alpha_ratio_mean** | **-0.12** | **0.24** | **0.01** | **-** | **-** | **0.3** | **0.77** | **-0.03** | **0.06** | **0.77** | **-** | **+** | **0.3** | **0.77** |
|  | alpha_ratio_sd | -0.07 | 0.14 | 0.14 | - | - | -0.37 | 0.71 | -0.03 | 0.07 | 0.74 | - | + | -0.37 | 0.71 |
|  | **average_mfccs_1** | **-0.16** | **0.31** | **0** | **-** | **-** | **1** | **0.32** | **0.1** | **0.2** | **0.09** | **+** | **+** | **1** | **0.32** |
|  | **average_mfccs_2** | **0.03** | **0.07** | **0.57** | **+** | **+** | **0.39** | **0.69** | **-0.16** | **0.32** | **0** | **-** | **-** | **0.39** | **0.69** |
|  | average_mfccs_3 | 0.07 | 0.13 | 0.18 | + | + | -1.14 | 0.25 | -0.05 | 0.11 | 0.46 | - | - | -1.14 | 0.25 |
|  | **average_mfccs_4** | **0.09** | **0.19** | **0.03** | **-** | **+** | **-2.68** | **0.01** | **-0.07** | **0.13** | **0.3** | **-** | **-** | **-2.68** | **0.01** |
|  | f1_relative_energy_mean | 0.01 | 0.03 | 0.8 | + | - | 1.36 | 0.17 | 0.07 | 0.14 | 0.3 | + | + | 1.36 | 0.17 |
|  | **f1_relative_energy_sd** | **-0.1** | **0.2** | **0.02** | **-** | **-** | **0.63** | **0.53** | **-0.06** | **0.13** | **0.3** | **-** | **-** | **0.63** | **0.53** |
|  | f2_relative_energy_mean | 0.04 | 0.08 | 0.52 | + | + | 0 | 1 | 0.08 | 0.16 | 0.23 | + | + | 0 | 1 |
|  | f2_relative_energy_sd | -0.01 | 0.03 | 0.8 | - | + | -0.76 | 0.45 | -0.01 | 0.02 | 0.91 | - | + | -0.76 | 0.45 |
|  | **f3_relative_energy_mean** | **0.11** | **0.23** | **0.01** | **+** | **+** | **-1.22** | **0.22** | **0** | **0** | **1** | **+** | **-** | **-1.22** | **0.22** |
|  | f3_relative_energy_sd | 0 | 0.01 | 0.96 | - | + | -1.43 | 0.15 | -0.01 | 0.03 | 0.9 | + | - | -1.43 | 0.15 |
|  | h1_a3_harmonic_difference_mean | 0.08 | 0.16 | 0.08 | + | + | -2.01 | 0.04 | -0.01 | 0.02 | 0.91 | + | - | -2.01 | 0.04 |
|  | h1_a3_harmonic_difference_sd | 0.02 | 0.03 | 0.8 | + | + | -0.16 | 0.87 | -0.03 | 0.05 | 0.79 | - | - | -0.16 | 0.87 |
|  | h1_h2_harmonic_difference_mean | 0.04 | 0.08 | 0.52 | + | + | -0.35 | 0.73 | -0.04 | 0.07 | 0.72 | - | + | -0.35 | 0.73 |
|  | h1_h2_harmonic_difference_sd | 0.05 | 0.09 | 0.41 | + | + | 0.53 | 0.6 | -0.05 | 0.09 | 0.55 | - | - | 0.53 | 0.6 |
|  | **hammarberg_index_mean** | **-0.12** | **0.24** | **0.01** | **-** | **-** | **2.89** | **0** | **0.02** | **0.03** | **0.9** | **-** | **+** | **2.89** | **<0.001** |
|  | hammarberg_index_sd | 0.04 | 0.09 | 0.41 | + | + | -0.58 | 0.56 | -0.01 | 0.03 | 0.91 | + | - | -0.58 | 0.56 |
|  | spectral_slope_0_500_mean | -0.04 | 0.07 | 0.56 | + | - | 2.3 | 0.02 | 0.09 | 0.18 | 0.11 | + | + | 2.3 | 0.02 |
|  | spectral_slope_0_500_sd | 0.05 | 0.1 | 0.37 | + | + | -0.79 | 0.43 | -0.04 | 0.07 | 0.72 | - | - | -0.79 | 0.43 |
|  | spectral_slope_500_1500_mean | 0.08 | 0.15 | 0.11 | + | + | -1.97 | 0.05 | -0.02 | 0.03 | 0.9 | - | + | -1.97 | 0.05 |
|  | spectral_slope_500_1500_sd | 0.02 | 0.04 | 0.79 | + | + | -0.13 | 0.9 | -0.01 | 0.01 | 0.91 | + | - | -0.13 | 0.9 |
| Temporal | duration | -0.02 | 0.04 | 0.79 | - | - | -0.13 | 0.9 | -0.04 | 0.08 | 0.72 | - | - | -0.13 | 0.9 |
|  | number_of_pauses | 0.02 | 0.04 | 0.79 | + | + | -0.12 | 0.91 | 0.03 | 0.06 | 0.77 | + | + | -0.12 | 0.91 |
|  | pause_durations_mean | -0.07 | 0.14 | 0.16 | - | - | 1.89 | 0.06 | -0.05 | 0.09 | 0.55 | - | - | 1.89 | 0.06 |
|  | pause_durations_sd | -0.06 | 0.12 | 0.22 | - | - | 1.77 | 0.08 | -0.02 | 0.03 | 0.9 | - | + | 1.77 | 0.08 |
|  | pause_durations_sum | -0.02 | 0.05 | 0.7 | + | - | 1.23 | 0.22 | -0.02 | 0.04 | 0.83 | - | + | 1.23 | 0.22 |
|  | pause_rate | -0.03 | 0.06 | 0.6 | + | - | 2.22 | 0.03 | -0.01 | 0.02 | 0.91 | - | + | 2.22 | 0.03 |
|  | speech_ratio | 0.02 | 0.03 | 0.79 | - | + | -3.61 | 0 | 0.07 | 0.14 | 0.3 | + | + | -3.61 | <0.001 |
|  | utterance_durations_mean | -0.02 | 0.04 | 0.79 | - | + | -2.35 | 0.02 | -0.02 | 0.05 | 0.79 | - | - | -2.35 | 0.02 |
|  | utterance_durations_sd | 0 | 0 | 0.98 | - | + | -2.01 | 0.04 | -0.01 | 0.02 | 0.91 | - | + | -2.01 | 0.04 |
|  | utterance_durations_sum | 0 | 0 | 0.98 | - | + | -2.09 | 0.04 | 0.01 | 0.01 | 0.91 | - | + | -2.09 | 0.04 |

**Supp. Table 4:** Correlations of acoustic features with STAXI scores across conditions. The table presents correlation coefficients (corr), effect sizes (ES), and adjusted p-values (p-adj) for the overall population. Sex-specific correlations are indicated separately for males (M) and females (F), with positive (+) or negative (-) directions. Fisher’s Z-test results, including Z-values and corresponding p-values, are reported under the "Sex Comparison" header to assess differences between sexes. Results are shown for both cold and warm conditions.

| **STAXI Difference Exposure to Stress Condition** | | **COLD** | | | | | | | **WARM** | | | | | | |
| --- | --- | --- | --- | --- | --- | --- | --- | --- | --- | --- | --- | --- | --- | --- | --- |
|  |  | ***Overall*** | | | ***Sex Comparison*** | | | | ***Overall*** | | | ***Sex Comparison*** | | | |
| **Feature Type** | **Feature** | **Corr** | **ES** | **p-adj** | **M** | **F** | **Z** | **p** | **Corr** | **ES** | **p-adj** | **M** | **F** | **Z** | **p** |
| Energy | apq3_shimmer | 0.02 | 0.04 | 0.69 | - | + | -0.51 | 0.61 | 0.04 | 0.08 | 0.62 | + | - | 1.42 | 0.16 |
|  | apq5_shimmer | 0.04 | 0.09 | 0.48 | + | + | 0.15 | 0.88 | 0.01 | 0.03 | 0.87 | + | - | 1.29 | 0.2 |
|  | dda_shimmer | 0.02 | 0.04 | 0.69 | - | + | -0.51 | 0.61 | 0.04 | 0.08 | 0.62 | + | - | 1.42 | 0.16 |
|  | **hnr_mean** | **-0.09** | **0.18** | **0.08** | **-** | **-** | **-0.65** | **0.52** | **0.14** | **0.28** | **0** | **+** | **+** | **1.09** | **0.28** |
|  | hnr_sd | 0.09 | 0.18 | 0.08 | + | + | 1.75 | 0.08 | -0.09 | 0.18 | 0.15 | - | - | 0.21 | 0.83 |
|  | local_dB_shimmer | -0.02 | 0.04 | 0.69 | - | + | -1.15 | 0.25 | 0.04 | 0.09 | 0.62 | + | - | 2.04 | 0.04 |
|  | local_shimmer | -0.02 | 0.03 | 0.72 | - | + | -0.77 | 0.44 | 0.02 | 0.04 | 0.81 | + | - | 2.21 | 0.03 |
|  | loudness_mean | -0.03 | 0.06 | 0.59 | - | - | 0.45 | 0.65 | 0 | 0.01 | 0.95 | - | + | -3.21 | <0.001 |
|  | loudness_sd | 0.08 | 0.17 | 0.08 | + | + | -0.96 | 0.34 | -0.02 | 0.04 | 0.81 | - | + | -0.96 | 0.34 |
|  | rate_loudness_peaks | -0.02 | 0.04 | 0.69 | - | + | -1.45 | 0.15 | 0.04 | 0.07 | 0.63 | + | + | -0.5 | 0.62 |
| Frequency | ddp_jitter | -0.04 | 0.08 | 0.48 | - | - | 0.15 | 0.88 | 0.1 | 0.19 | 0.11 | + | + | 0.69 | 0.49 |
|  | f1_bandwidth_mean | 0.01 | 0.02 | 0.83 | - | + | -0.82 | 0.41 | 0 | 0.01 | 0.95 | - | + | -0.82 | 0.41 |
|  | f1_bandwidth_sd | 0.07 | 0.14 | 0.17 | + | + | 0.53 | 0.6 | -0.03 | 0.06 | 0.73 | - | - | 0.31 | 0.76 |
|  | f1_frequency_mean | 0.03 | 0.05 | 0.66 | - | + | -1.58 | 0.11 | -0.06 | 0.12 | 0.48 | - | - | -0.13 | 0.9 |
|  | f1_frequency_sd | 0.04 | 0.09 | 0.48 | + | + | -0.47 | 0.64 | -0.03 | 0.05 | 0.8 | + | - | 1.49 | 0.14 |
|  | f2_bandwidth_mean | 0.03 | 0.06 | 0.57 | + | + | 0.57 | 0.57 | -0.08 | 0.16 | 0.18 | - | - | -0.49 | 0.63 |
|  | f2_bandwidth_sd | 0.04 | 0.08 | 0.48 | + | + | -0.06 | 0.95 | -0.02 | 0.04 | 0.81 | - | - | -0.28 | 0.78 |
|  | f2_frequency_mean | 0.04 | 0.08 | 0.49 | - | + | -1.71 | 0.09 | -0.03 | 0.06 | 0.72 | - | - | -0.14 | 0.89 |
|  | f2_frequency_sd | 0.08 | 0.17 | 0.08 | + | + | 1.25 | 0.21 | -0.04 | 0.08 | 0.62 | - | - | -0.19 | 0.85 |
|  | f3_bandwidth_mean | -0.06 | 0.13 | 0.25 | + | - | 2.48 | 0.01 | 0.04 | 0.07 | 0.63 | - | + | -1.21 | 0.23 |
|  | f3_bandwidth_sd | 0.03 | 0.05 | 0.66 | + | + | 0.67 | 0.5 | -0.04 | 0.08 | 0.62 | - | - | -0.35 | 0.73 |
|  | f3_frequency_mean | -0.04 | 0.08 | 0.48 | + | - | 1.37 | 0.17 | -0.08 | 0.16 | 0.18 | - | - | -0.66 | 0.51 |
|  | f3_frequency_sd | -0.08 | 0.17 | 0.08 | - | - | 1.51 | 0.13 | 0.04 | 0.08 | 0.62 | + | - | 1.4 | 0.16 |
|  | local_absolute_jitter | -0.05 | 0.1 | 0.48 | - | - | 1.19 | 0.24 | 0.08 | 0.15 | 0.2 | + | + | 0.46 | 0.65 |
|  | local_jitter | -0.04 | 0.07 | 0.53 | - | - | 0.66 | 0.51 | 0.08 | 0.16 | 0.18 | + | + | 0.8 | 0.43 |
|  | pitch_max | 0.08 | 0.17 | 0.08 | + | + | 0.73 | 0.46 | -0.02 | 0.04 | 0.81 | - | - | 0.33 | 0.74 |
|  | pitch_mean | 0.02 | 0.04 | 0.69 | - | + | -0.8 | 0.42 | -0.01 | 0.02 | 0.92 | + | - | 0.15 | 0.88 |
|  | pitch_min | -0.05 | 0.1 | 0.48 | - | - | -1.04 | 0.3 | 0 | 0 | 0.96 | - | + | -2.52 | 0.01 |
|  | pitch_range | 0.09 | 0.19 | 0.08 | + | + | 1.33 | 0.19 | -0.05 | 0.11 | 0.51 | + | - | 2.23 | 0.03 |
|  | pitch_std | 0.08 | 0.17 | 0.08 | + | + | 1.6 | 0.11 | -0.03 | 0.05 | 0.8 | + | - | 1.24 | 0.22 |
|  | **ppq5_jitter** | **-0.04** | **0.08** | **0.48** | **-** | **-** | **0.06** | **0.95** | **0.14** | **0.29** | **0** | **+** | **+** | **0.41** | **0.68** |
|  | rap_jitter | -0.04 | 0.08 | 0.48 | - | - | 0.15 | 0.88 | 0.1 | 0.19 | 0.11 | + | + | 0.69 | 0.49 |
|  | vocal_tremor | -0.02 | 0.04 | 0.69 | - | + | -0.93 | 0.35 | 0.02 | 0.04 | 0.81 | + | - | 0.51 | 0.61 |
| Spectral | alpha_ratio_mean | -0.07 | 0.14 | 0.17 | + | - | 2.6 | 0.01 | 0.04 | 0.08 | 0.62 | + | - | 1.56 | 0.12 |
|  | alpha_ratio_sd | -0.04 | 0.07 | 0.53 | + | - | 2.03 | 0.04 | 0.02 | 0.05 | 0.81 | + | + | 0.46 | 0.65 |
|  | average_mfccs_1 | -0.05 | 0.11 | 0.45 | + | - | 1.99 | 0.05 | 0 | 0.01 | 0.95 | - | + | -0.83 | 0.41 |
|  | average_mfccs_2 | -0.03 | 0.06 | 0.59 | + | - | 3.06 | 0 | -0.03 | 0.06 | 0.73 | - | - | 0.79 | 0.43 |
|  | average_mfccs_3 | 0.02 | 0.04 | 0.69 | - | + | -3.12 | 0 | -0.01 | 0.02 | 0.92 | + | - | 1.85 | 0.06 |
|  | average_mfccs_4 | -0.04 | 0.09 | 0.48 | - | + | -4.79 | 0 | 0.08 | 0.16 | 0.18 | + | - | 2.19 | 0.03 |
|  | f1_relative_energy_mean | 0.02 | 0.04 | 0.69 | + | - | 1.23 | 0.22 | -0.02 | 0.04 | 0.81 | - | - | 0.11 | 0.91 |
|  | f1_relative_energy_sd | -0.05 | 0.1 | 0.48 | - | - | -0.57 | 0.57 | 0.05 | 0.09 | 0.62 | + | - | 2.04 | 0.04 |
|  | f2_relative_energy_mean | 0.04 | 0.08 | 0.48 | - | + | -1.94 | 0.05 | 0 | 0.01 | 0.95 | - | + | -0.51 | 0.61 |
|  | f2_relative_energy_sd | -0.02 | 0.04 | 0.69 | + | - | 1.06 | 0.29 | -0.05 | 0.11 | 0.51 | - | - | -0.93 | 0.35 |
|  | f3_relative_energy_mean | 0.12 | 0.24 | 0.02 | + | + | -3.31 | 0 | -0.08 | 0.16 | 0.18 | - | - | -0.4 | 0.69 |
|  | f3_relative_energy_sd | -0.01 | 0.02 | 0.78 | + | - | 1.23 | 0.22 | -0.01 | 0.01 | 0.95 | - | - | -0.08 | 0.93 |
|  | h1_a3_harmonic_difference_mean | 0.12 | 0.23 | 0.02 | - | + | -3.35 | 0 | -0.02 | 0.05 | 0.81 | + | - | 0.62 | 0.53 |
|  | h1_a3_harmonic_difference_sd | 0 | 0.01 | 0.93 | + | - | 0.96 | 0.34 | -0.02 | 0.05 | 0.81 | + | - | 1.65 | 0.1 |
|  | h1_h2_harmonic_difference_mean | 0 | 0.01 | 0.9 | - | + | -0.92 | 0.36 | -0.05 | 0.11 | 0.51 | - | + | -1.49 | 0.14 |
|  | **h1_h2_harmonic_difference_sd** | **0.1** | **0.2** | **0.05** | **+** | **+** | **-0.77** | **0.44** | **0.02** | **0.04** | **0.81** | **-** | **+** | **-0.69** | **0.49** |
|  | **hammarberg_index_mean** | **-0.13** | **0.27** | **0** | **+** | **-** | **4.62** | **0** | **0.09** | **0.19** | **0.12** | **+** | **+** | **0.41** | **0.69** |
|  | hammarberg_index_sd | 0.05 | 0.09 | 0.48 | + | + | 1.52 | 0.13 | -0.04 | 0.09 | 0.62 | - | - | 0.4 | 0.69 |
|  | spectral_slope_0_500_mean | -0.02 | 0.03 | 0.7 | + | - | 1.49 | 0.14 | 0 | 0.01 | 0.95 | - | + | -0.99 | 0.32 |
|  | spectral_slope_0_500_sd | 0.03 | 0.06 | 0.63 | + | + | -0.15 | 0.88 | 0.02 | 0.04 | 0.81 | + | + | 0.28 | 0.78 |
|  | spectral_slope_500_1500_mean | -0.02 | 0.03 | 0.7 | - | - | -0.03 | 0.98 | 0.05 | 0.09 | 0.62 | + | + | -0.9 | 0.37 |
|  | spectral_slope_500_1500_sd | 0.02 | 0.04 | 0.69 | + | + | -0.35 | 0.73 | -0.06 | 0.11 | 0.51 | - | - | 0.61 | 0.54 |
| Temporal | duration | 0.04 | 0.09 | 0.48 | + | + | 0.32 | 0.75 | -0.03 | 0.06 | 0.73 | - | - | -0.15 | 0.88 |
|  | number_of_pauses | 0.04 | 0.08 | 0.48 | + | + | 0.99 | 0.32 | 0.02 | 0.03 | 0.87 | + | - | 0.62 | 0.53 |
|  | pause_durations_mean | 0.04 | 0.08 | 0.48 | + | + | -0.23 | 0.82 | -0.04 | 0.07 | 0.67 | - | - | 0.17 | 0.87 |
|  | pause_durations_sd | 0.04 | 0.08 | 0.48 | + | + | 0.38 | 0.7 | -0.02 | 0.03 | 0.82 | + | - | 0.85 | 0.4 |
|  | pause_durations_sum | 0.06 | 0.13 | 0.25 | + | + | 0.35 | 0.73 | -0.01 | 0.03 | 0.87 | + | - | 0.72 | 0.47 |
|  | pause_rate | 0.03 | 0.07 | 0.54 | + | + | 0.16 | 0.87 | 0.01 | 0.02 | 0.9 | + | - | 1.26 | 0.21 |
|  | speech_ratio | -0.04 | 0.08 | 0.48 | - | - | -0.96 | 0.34 | 0.06 | 0.11 | 0.51 | + | + | 1.17 | 0.24 |
|  | utterance_durations_mean | -0.02 | 0.04 | 0.69 | - | + | -0.95 | 0.34 | -0.01 | 0.03 | 0.87 | - | - | -0.03 | 0.98 |
|  | utterance_durations_sd | 0.01 | 0.02 | 0.78 | - | + | -1.63 | 0.1 | -0.04 | 0.09 | 0.62 | - | - | -0.13 | 0.9 |
|  | utterance_durations_sum | 0.02 | 0.05 | 0.66 | + | + | -0.38 | 0.7 | 0 | 0.01 | 0.95 | + | - | 0.78 | 0.44 |

**Supp. Table 5:** Significant associations between speech features and predictor variables from linear regression models. The models were adjusted for changes in cortisol levels (Cortisol_change), baseline cortisol levels (Cortisol_before), and sex (sex_m). Effect sizes (β) are presented with their corresponding p-values, standard errors (SE), effect direction (↑ for positive, ↓ for negative). Only results with *p* values<0.05 are shown.

| **Predictor Variable** | **Speech Feature** | **Effect Size (β)** | **Effect Direction** | **P-Value** | **Standard Error (SE)** |
| --- | --- | --- | --- | --- | --- |
| Cortisol_before | average_mfccs_3 | -0.61 | ↓ | 0.014 | 0.25 |
|  | local_shimmer | -0.2 | ↓ | 0.003 | 0.07 |
| Cortisol_change | alpha_ratio_mean | -0.05 | ↓ | 0.02 | 0.02 |
| sex_m | apq3_shimmer | -0.83 | ↓ | 0.016 | 0.35 |
|  | dda_shimmer | -2.5 | ↓ | 0.016 | 1.04 |
|  | ddp_jitter | -0.55 | ↓ | 0.026 | 0.25 |
|  | local_jitter | -0.28 | ↓ | 0.026 | 0.12 |
|  | local_shimmer | -1.16 | ↓ | 0.028 | 0.53 |
|  | rap_jitter | -0.18 | ↓ | 0.026 | 0.08 |
|  | pitch_std | -3.59 | ↓ | 0.016 | 1.49 |

**Supp. Table 6:** Machine Learning (ML) results. AUC=Area under the curve; DT=Decision Trees; k=number of features included in respective model; LM=Linear Model; MAE=Mean Absolute Error; RF=Random Forests; SVM=Support Vector Machine; XT=Extra Trees.

| **Model** | **Task** | **k** | **Score** |
| --- | --- | --- | --- |
| SVM | Classification Cortisol (AUC) | 15 | 0.55 |
| XT | Classification Cortisol (AUC) | 15 | 0.54 |
| RF | Classification Cortisol (AUC) | 15 | 0.53 |
| LM | Classification Cortisol (AUC) | 40 | 0.53 |
| DT | Classification Cortisol (AUC) | 5 | 0.51 |
| SVM | Regression Cortisol (MAE) | 5 | 3.82 |
| XT | Regression Cortisol (MAE) | 25 | 4.57 |
| RF | Regression Cortisol (MAE) | 25 | 4.65 |
| LM | Regression Cortisol (MAE) | 5 | 4.43 |
| DT | Regression Cortisol (MAE) | 15 | 6.04 |
| SVM | Regression STAXI (MAE) | 5 | 1.36 |
| XT | Regression STAXI (MAE) | 50 | 1.69 |
| RF | Regression STAXI (MAE) | 40 | 1.75 |
| LM | Regression STAXI (MAE) | 5 | 1.56 |
| DT | Regression STAXI (MAE) | 45 | 2.18 |

**Supp. Table 7:** Number of times the ten most selected speech features were included in machine learning classification and regression models.

| **Speech Feature** | **Classification** | **Cortisol Regression** | **STAXI Regression** | **Total** |
| --- | --- | --- | --- | --- |
| average_mfccs_3 | 5 | 5 | 5 | 15 |
| average_mfccs_2 | 5 | 5 | 5 | 15 |
| apq5_shimmer | 3 | 3 | 3 | 9 |
| f1_bandwidth_mean | 3 | 3 | 3 | 9 |
| average_mfccs_4 | 3 | 3 | 3 | 9 |
| f1_frequency_mean | 3 | 3 | 3 | 9 |
| duration | 3 | 3 | 3 | 9 |
| alpha_ratio_mean | 3 | 3 | 3 | 9 |
| f1_bandwidth_sd | 2 | 2 | 2 | 6 |
| spectral_slope_500_1500_mean | 2 | 2 | 2 | 6 |
